# Beyond sound irritation: cross-cultural evidence on the robustness of the five aspects of misophonic experience measured by the S-Five in a Polish sample

**DOI:** 10.3389/fpsyg.2024.1372870

**Published:** 2024-06-19

**Authors:** Nora Uglik-Marucha, Marta Siepsiak, Julia Zielińska, Wojciech Łukasz Dragan, Jane Gregory, Silia Vitoratou

**Affiliations:** ^1^Psychometrics and Measurement Lab, Biostatistics and Health Informatics Department, Psychology and Neuroscience, King’s College London, London, United Kingdom; ^2^Faculty of Psychology, University of Warsaw, Warsaw, Poland; ^3^Institute of Psychology, Jagiellonian University, Kraków, Poland; ^4^Department of Experimental Psychology, University of Oxford, Oxford, United Kingdom; ^5^Oxford Health NHS Foundation Trust, Oxford, United Kingdom

**Keywords:** misophonia, psychometrics, S-Five, polish, measurement

## Abstract

Misophonia is commonly associated with negative emotional or physiological responses to specific sounds. However, the consensus definition emphasizes that misophonia entails much more than that. Even in cases of subclinical misophonia, where individuals do not meet the disorder criteria, the experience can still be burdensome, despite not currently causing significant distress or impairment. The S-Five is a psychometric tool for comprehensive assessment of five aspects of misophonic experience: internalizing, externalizing, impact, threat, and outburst, and includes S-Five-T section to evaluate feelings evoked by triggering sounds and their intensity. We examined whether the five-factor structure developed in the UK could be replicated in a Polish sample, including individuals with and without self-identified misophonia. The Polish version of the S-Five was translated and tested on 288 Polish-speaking individuals. Comprehensive psychometric evaluation, including factor structure, measurement invariance, test–retest reliability, internal consistency, and concurrent validity evaluations, was conducted on the translated scale. Exploratory factor analysis suggested similar structure to the original English study, while bootstrap exploratory graph analysis showed the factor structure to be reproducible in other samples. The scale was found to be bias free with respect to gender, internally consistent and stable in time, and evidence of validity was provided using MisoQuest and Misophonia Questionnaire. These results offer support for the cross-cultural stability of the five factors and provide preliminary evidence for the suitability of the Polish version for clinical and research purposes. The study also investigated five facets of misophonia, triggering sounds, emotional responses, and their associations with symptoms of psychopathology across various cultures. It underscores the central role of anger, distress, and panic, while also highlighting the mixed role of irritation and disgust in misophonia across different cultural contexts. Mouth sounds evoked the most pronounced reactions compared to other repetitive sounds, although there were discernible cultural differences in the nature and intensity of reactions to various trigger sounds. These findings hold significant implications for future research and underscore the importance of considering cultural nuances in both research and the clinical management of misophonia.

## Introduction

Misophonia is a recently recognized disorder characterized by reduced tolerance to specific sounds or stimuli (Jastreboff and Jastreboff, 2002; Swedo et al., 2022). These sounds, known as ‘triggers,’ can elicit intense negative emotional, behavioral, and physiological responses unique to each individual (Edelstein et al., 2013; Kumar et al., 2017; Brout et al., 2018) and may include anger, disgust, irritation, and anxiety (Jager et al., 2020; Swedo et al., 2022). Physiological reactions such as increased heart rate, muscular tension, and sweating can also occur (Edelstein et al., 2013; Rouw and Erfanian, 2018; Schröder et al., 2019). Interestingly, misophonia’s manifestation can be context-dependent (for instance, Heller and Smith, 2022; Samermit et al., 2022) with reactions being more pronounced or limited to triggers produced by close friends and family (Edelstein et al., 2013). A recent study by Siepsiak et al. (2023b) further revealed that the context of mouth sounds can significantly influence affective experiences in adults with misophonia. Misophonia can profoundly impact an individual’s life, affecting work, academics, and interpersonal relationships (Brout et al., 2018).

In recent years, the literature on misophonia has seen a surge in interest, with more scientific articles being published between the years 2020 and 2023 than in all the previous years combined. Neacsiu et al. (2022) conducted a literature review on the neurobiological basis of misophonia, concluding that it has unique neurobiological features that distinguish it from other disorders. Consistent with this finding, studies have shown that misophonia frequently co-occurs with various psychiatric disorders and symptoms of psychopathology (Erfanian et al., 2019; Jager et al., 2020; Rosenthal et al., 2022; Siepsiak et al., 2022), even in childhood (Rinaldi et al., 2022; Guzick et al., 2023; Siepsiak et al., 2023a). However, there is no specific diagnostic entity that can be identified as exclusively characteristic of misophonia or that can fully explain its unique symptoms.

The prevalence of misophonia is currently estimated to vary between 5 to 20% in different populations. Studies based on representative samples of Germany, Turkey and the UK populations reported that 5, 12.8, and 18.4% of people experience burdensome symptoms of misophonia (Kılıç et al., 2021; Jakubovski et al., 2022; Vitoratou et al., 2023). However, these studies have not identified a cut-off at which symptoms would be considered a disorder, and therefore these rates may include those experiencing subclinical symptoms, which may be burdensome to the individual but are not currently causing significant distress and impairment (Williams et al., 2022). Similar rates (6–20%) have been mentioned in student populations (Wu et al., 2014; Zhou et al., 2017; Sarigedik and Gulle, 2021) and a clinical sample (9–12%; Siepsiak et al., 2020a), while one study on medical students revealed a surprisingly high occurrence of this disorder in almost 50% of participants when mild symptoms were included in the threshold (Naylor et al., 2021). Although various factors may contribute to the significant differences observed in the outcomes of these studies, it is crucial to employ cross-culturally validated questionnaires with rigorous psychometric properties. This approach ensures a more accurate measurement of misophonia and facilitates meaningful comparisons across different countries and populations. In a systematic review, Kula et al. (2023) examined psychometric measures of misophonia published in English between 2002 and 2020. Notably, these tools were utilized in numerous studies establishing the prevalence of misophonia. However, the measures have shown limitations in meeting the requirements of the Consensus-based Standards for the selection of health status Measurement Instruments (COSMIN) (Mokkink et al., 2010a,b). This highlights the necessity for improved and more comprehensive tools to accurately measure and assess misophonia symptoms. More recently, new self-report measurement tools have been developed, including the Duke-Vanderbilt Misophonia Screening Questionnaire (Williams et al., 2022), the Duke Misophonia Questionnaire (Rosenthal et al., 2021), and the S-Five (Vitoratou et al., 2021a), offering promising alternatives for future research and prevalence studies.

In this work we focus on the S-Five. To assess the complex experience of misophonia and its severity, Vitoratou et al. (2021b) developed the Selective Sound Sensitivity Syndrome Scale (S-Five) using the responses of English-speaking population self-identifying with misophonia in four waves of sampling. The S-Five identified five aspects of misophonic experience, namely (i) externalizing appraisals, which represents the feelings of blame and judgement for experiencing negative responses to sounds that are directed towards other people, (ii) internalizing appraisals, when the judgement and blame is directed towards oneself, (iii) impact, which describes perceived restrictions to functioning due to reactions to sounds, (iv) threat, feeling of distress (about emotions escalating) around triggering sounds, and (v) outburst, which include the presence or fear of having physical or verbal explosion in response to some sounds. The questionnaire underwent further psychometric testing and was shown to have satisfactory psychometric properties with respect to reliability (stability and internal consistency), convergent validity, and measurement invariance. With respect to the responses to triggering sounds, the S-Five comes with a supplementary checklist, the S-Five-T, which allows for investigations of specific reactions and their intensity to certain sounds. For instance, Vitoratou et al. (2021a) reported that people self-identifying with misophonia were more than 40 and 20 times more likely than those without misophonia to be triggered by eating sounds and breathing sounds, respectively. The S-Five and the S-Five-T can also be used to investigate mechanisms and response to change to intervention or treatment with respect to the five dimensions of misophonia. Incorporated into research, it can provide insight into understanding the correlates of specific facets of misophonia with other conditions or into longitudinal changes in the severity of symptoms. The five-factor structure of the S-Five was further replicated in a large sample representative of the UK population, demonstrating excellent psychometric properties (Vitoratou et al., 2023). The robustness of the factor structure of the S-Five is further evidenced through its replications across different languages and cultures, namely in German (Remmert et al., 2022), Chinese (Vitoratou et al., 2022) and Portuguese (Hayes et al., 2024) populations.

There have been only two measures of misophonia available in the Polish language, namely MisoQuest (Siepsiak et al., 2020b) and Misophonia Questionnaire (MQ; Wu et al., 2014). MisoQuest was the first scale in misophonia literature with published full psychometric evaluation in a peer-reviewed journal, whose properties were assessed in the Polish population. MisoQuest is a unidimensional measure for identifying the presence of misophonia defined according to a very specific and narrow criteria, developed by Schröder et al. (2013) [which were later revised by Jager et al. (2020)], with minor modifications applied by authors. For instance, the diagnostic criteria for misophonia by Schröder et al. (2017) include the presence of spontaneous and aversive reaction to sounds produced by humans only, while MisoQuest assesses for the presence of all ranges of sounds, both human and non-human. The scale has demonstrated good validity and reliability (Siepsiak et al., 2020b), very high specificity but limited sensitivity (Siepsiak et al., 2020b; Enzler et al., 2021). Importantly, it was neither developed to measure the severity of misophonia symptoms in individuals with misophonia, nor developed using a multidimensional reflective latent variables model. Furthermore, the MisoQuest detects the presence of misophonia when the aversive emotional response is immediate. Individuals who only react negatively to a prolonged exposure to external stimuli would probably not be identified; however, those diagnosed would constitute a more homogenous group. Recently its psychometric properties were evaluated in Canada (Raymond, 2023), UK (Kula et al., 2024), and Turkey (Ay et al., 2024). MQ was developed to assess misophonia symptoms in three sections, investigating the symptoms sensitivity (MSYS), person’s behaviors and emotions (MEBS), and the severity of the symptoms (MEBS). MQ has been translated to Polish but its psychometric properties have not yet been evaluated and published. The original English version has shown satisfactory internal consistency (Wu et al., 2014). Although the full psychometric properties of the English scale have not been published, it has been recently validated for use in Norway (Larsen et al., 2023), Turkey (Sakarya and Çakmak, 2022) and Iran (Mehrabizadeh Honarmand and Roushani, 2019). Despite this, MQ has been the most widely used questionnaire for the assessment of misophonia (Zhou et al., 2017; Cusack et al., 2018; Janik McErlean and Banissy, 2018; McKay et al., 2018). Notably, the MSES, which is frequently used as a cut-off score to create groups with and without misophonia, was developed on the basis of items assessing obsessive-compulsive disorder severity and, to our knowledge, has not been validated as a tool to discriminate between those with and without misophonia.

In Poland, the notable barrier in misophonia research has been a limited possibility to compare the data collected in the Polish population with the data collected by other researchers across the world. Furthermore, to our knowledge, there has been no measurement tool available in the Polish language for a multidimensional assessment of the severity of the misophonic experience at the time the study was conducted. In light of this, the aim of this study was to translate the S-Five into Polish language and validate it in the population of Polish-speaking individuals, both those who self-identify with having the condition and those who do not. The Polish version of the S-Five would supplement the assessment of misophonia in Polish-speaking populations in both therapeutic and research context and address the barriers in cross-cultural research of misophonia by allowing for international investigations.

In accordance with the findings by Vitoratou et al. (2021b) in English-speaking population, it was hypothesized that:

1. The S-Five will show a five-factor structure in the Polish sample,
2. The S-Five will demonstrate satisfactory psychometric properties with respect to internal consistency, test–retest reliability, measurement invariance with respect to gender adjusted for age, and convergent validity with MisoQuest and MQ and its subscales (except for MSYS),
3. The S-Five will emerge weakly to moderately correlated with depression and anxiety.

A secondary aim of the study was to explore relationships between S-Five factors, reactions to triggers, symptoms of anxiety and depression, and traits of anxiety sensitivity. We did not have any hypotheses for this aim, and planned to discuss these relationships in relation to similar studies in other languages and cultural groups.

## Methods

### Study overview

Participants were recruited between December 2021 and March 2022 through groups on social media sites relating to misophonia, namely Facebook (‘Mizofonicy’ group), Reddit (r/misophonia), and Twitter (#misophonia, #mizofonia). The survey was administered online via Qualtrics. Before proceeding with the study, participants were provided with a participants’ information sheet and consent of participation was established (ethics approval reference RESCM-19/20–11,826). Inclusion criteria for participation included being aged 18 years and over, as the original scale has only been validated in adult populations, and Polish fluency. Participants who indicated the presence of severe intellectual and/or learning disability at the beginning of the survey were automatically excluded from continuing with the study. At the end of the survey, participants were asked about the participation in a retest study, which involved entering their e-mail addresses in a separate survey to avoid linking their responses with their contact information. Those who agreed to participate in the retest survey were contacted again 3 weeks after the initial assessment. An Amazon voucher of £20 pounds was offered for one in 20 participants as a lucky draw to encourage participation.

The online survey included the S-Five and other measures for validity and hypothesis testing purposes (please see below for details on the battery used). The items of S-Five and the blocks of the remaining questionnaires were presented to the participants in random order. Throughout the survey, attention check questions were administered allowing for screening of the data for low-quality responses. Demographic characteristics were collected, such as age, gender, occupation, education level, and country of birth and residence. Participants were asked to report any formal diagnoses they may have received of mental health conditions (affective, anxiety, personality, trauma, psychotic, substance abuse and eating disorders), neurodevelopmental conditions (autism, dyslexia, ADHD), and audiological conditions (hyperacusis, tinnitus, auditory processing disorder).

### Translation process

The translation process followed a framework outlined by the Oxford University Innovation (Wild et al., 2005). This process took the following steps:

1. Forward translation of the English version of the S-Five into Polish was performed simultaneously by two independent researchers (NUM & JZ) to produce two translations. Both NUM and JZ are bilingual with Polish as their mother tongue. One of the translators (NUM) acted as a subject expert due to being a co-author of the original S-Five, thus ensuring that misophonia intricacies were not missed.
2. Reconciliation of forward translations was achieved by comparing and merging the two Polish versions by NUM and JZ. The discrepancies in translations were discussed and resolved with an independent bilingual translator with Polish as their native language (AT). This step resulted in a preliminary Polish version of the S-Five.
3. Back-translation of the Polish version of the S-Five to English was performed independently by two bilingual researchers (MS & WD) with Polish as their mother tongue, who were not involved in the previous exercise. The two researchers were subject experts on misophonia.
4. Reconciliation of back-translation was conducted by SV and JG, original developers of the S-Five, by comparing the two back-translated versions with the source text. In this step, minor discrepancies were identified with respect to 4 items, which were discussed between NUM, JZ, and MS to reach a consensus on a final version of items. This led to minor revisions, which were reported back to SV and JG for the approval, and thus resulting in the final translated Polish version of the S-Five.
5. Testing of the translated questionnaire was performed on the Polish population self-identifying with having misophonia. In the survey, a space was provided for the participants to provide feedback on the translated items.

### Measures

The **S-Five** (Vitoratou et al., 2021b) is a self-report scale which consists of 25 items that are rated on an 11-point scale ranging from 0 (not at all true) to 10 (completely true), with a total score range of 0–250. There are five factors with five items each, with a score range of 0–50 for each of the following domains: externalizing appraisals, internalizing appraisals, perceived emotional threat, outbursts, and impact on functioning. Please see Supplementary Table S1 for both English and Polish items and Supplementary material 2 for fillable form with automatic scoring in a pdf format.

A supplementary S-Five trigger checklist (S-Five-T) consists of 37 trigger sounds. Respondents select their main response to each sound from the following options: no feeling, irritation, distress, disgust, anger, panic, other feeling: negative, other feeling: positive, and physiological reaction. The latter option was recently added in the German validation of the S-Five (Remmert et al., 2022), and was also used in the present study. The intensity of the selected reaction is then rated on an 11-point scale from 0 (does not bother me at all) to 10 (unbearable/causes suffering). The checklist results in four summary indices: trigger count (TC; the number of triggers endorsed), reaction count (RC; the number of times each reaction is endorsed across all triggers), frequency/intensity of reactions score (FIRS; the total score for the intensity items across all endorsed triggers), and relative intensity of reactions score (RIRS; intensity of reactions relative to the number of triggers). The original and Polish items are presented in Supplementary Table S2.

The **MisoQuest** (Siepsiak et al., 2020b) is a self-report measure of misophonia validated in a sample of Polish-speaking participants that was developed around the diagnostic criteria proposed by Schröder et al. (2013). The questionnaire is a unidimensional tool consisting of 14 items, which are rated on a 1 to 5 agreement scale. In this study, MisoQuest had both Cronbach’s alpha (α) and McDonald’s omega (ω) of 0.91.

The **Misophonia Questionnaire** (MQ; Wu et al., 2014) is a self-report questionnaire consisting of 34 items that form three parts. The Misophonia Symptoms Scale (MSYS) measures the sensitivity to a specific trigger when compared to others, and the Misophonia Emotions and Behaviours Scale (MEBS) assesses for emotional and behavioral responses to trigger sounds. Both parts are rated on a 5-point ordinal scale, which are combined to form the MQ total score. The third part, the Misophonia Severity Scale (MSES) is a single-item question that measures the severity of sound sensitivity on a 15-point scale, where 1 indicates minimal and 15 suggests very severe sensitivity. A score of 7 or above on the MSES is proposed to indicate clinical levels of misophonia. The MQ was translated by MS as a part of another, yet unpublished study. In the present study, the subscales of MQ had both α and ω of 0.74 and 0.85 for MSYS and MEBS, respectively. Cronbach’s α for the total scale was 0.84 and ω was 0.83.

The **Patient Health Questionnaire-9** (PHQ-9; Kroenke et al., 2001) is a brief measure of the severity of depression. The tool includes 9 items that are rated on a 4-point ordinal scale that form a total score ranging from 0 to 27. PHQ-9 has been validated for the use in Polish, and such a version was used in this study (Kokoszka et al., 2016). In this study, the measure had an α and ω of 0.84.

The **Generalized Anxiety Disorder-7** (GAD-7; Spitzer et al., 2006), validated for use in Poland by Basińska and Kwissa-Gajewska (2023) screens and measures the severity of generalized anxiety disorder. GAD-7 has 7 items and uses a 4-point ordinal scale, which are summed to create a total score for all items (0 to 21). In this study, the GAD-7 demonstrated satisfactory internal consistency, with both α and ω of 0.83, respectively.

The **Anxiety Sensitivity Index-3** (ASI-3; Taylor et al., 2007) is a self-report measure of anxiety sensitivity, that is, concerns regarding anxiety symptoms. ASI-3 consists of 18 items forming 3 subscales, with responses rated on a 5-point scale ranging from 0 (very little) to 4 (very much). The three subscales measure the physical, cognitive, and social concerns about anxiety. All items are summed to create ASI-3 total (0 to 72) and the sum of items consisting of each subscale provides a subscale score (0 to 24). A Polish version that was validated in the Polish population was used in this study (Michałowski et al., 2014). The cognitive and physical subscales had both α and ω of 0.90 and 0.87, respectively and social subscale had an α and ω of 0.83 and 0.82, respectively. The entire measure demonstrated α and ω of 0.92.

### Statistical analysis

#### Dimensionality

Exploratory factor analysis (EFA) was used for evaluation of the structure of the scale, adhering to recommendations for adapting instruments to a new cultural context (Gjersing et al., 2010). Exploratory methods were also employed to investigate the presence of cross-loadings in the Polish version, which had previously emerged during the validation of the Mandarin version (Vitoratou et al., 2022). The amenability of a correlation matrix to factoring was evaluated using the Kaiser-Meyer-Olkin (KMO) measure of sampling adequacy (KMO of ≥0.90 suggests suitability; Kaiser, 1960; Kaiser and Rice, 1974) and the Bartlett’s test of sphericity (significant difference between the correlation matrix and the identity matrix indicates amenability to factoring; Bartlett, 1951). Mardia’s (1970) multivariate skewness and kurtosis coefficients were calculated, which indicated violation of multivariate normality (*p* < 0.001). To account for skewed continuous data, the maximum likelihood estimator with robust standard errors MLR was used (Muthén and Muthén, 1998–2012). Two common factor selection procedures were employed, the Kaiser-Guttman criterion (Guttman, 1954; Kaiser, 1960) and parallel analysis (Horn, 1965). The first suggests retaining the number of factors that correspond to the number of eigenvalues of the sample correlation matrix that are greater than one. Parallel analysis compares the number of sample eigenvalues with the mean of eigenvalues generated from 50 random samples of the same size and number of variables. The number of factors to be retained corresponds to the number of eigenvalues larger than the average eigenvalues from the random data.

The solutions suggested by the Kaiser-Guttman criterion and parallel analysis were compared with plus minus one factor solutions to evaluate which solution is most interpretable (Lim and Jahng, 2019). Several goodness of fit indices were used to evaluate proposed solutions. The absolute fit of the model was assessed using relative χ^2^, the root mean square error of approximation (RMSEA; Steiger, 1990; Browne and Cudeck, 1992) and its 90% confidence interval (90% CI), and the standardized root mean square residual (SRMR). Fit relative to a null model was evaluated using the comparative fit index (CFI; Bentler, 1990) and the Tucker–Lewis index (TLI; Tucker and Lewis, 1973). Guidelines for close fit provided in Hu and Bentler (1999) and Ullman (2006) were followed with the following criteria: relative χ^2^ (≤ 2), RMSEA (≤ 0.05, 90% CI ≤ 0.06), SRMR (≤ 0.08), CFI (≥ 0.95), and TLI (≥ 0.95).

Item selection was based on the items’ factor loadings, with loadings of at least 0.30 on the main factor identified as a meaningful indicator of a latent construct and to be retained (Brown, 2015). Items with loadings below 0.3 and/or cross-loadings on other factors (*λ* ≥ 0.25) were considered redundant.

To obtain accurate estimates for factor solutions, the ratio of the participants to the number of measured indicators was considered (Kyriazos, 2018). For this study, 25 items were used in dimensionality investigations, which requires 250 cases for EFA alone. The study’s sample (*N* = 288) was sufficient for EFA but did not allow for further dimensionality explorations using confirmatory factor analysis.

#### Exploratory graph analysis

In addition to those traditional factor reduction methods, a new method derived from the network psychometrics framework was conducted to assess the dimensionality underlying the data and to evaluate the stability of the established factor structure. Exploratory Graph Analysis (EGA) (Golino and Epskamp, 2017) was used to generate a visual guide-network plot that depicts relations between the items and factors. In these network models, nodes (circles) correspond to items, and the edges (lines) represent correlations between two items. The basis of the network structure is derived from a Gaussian Graphical Model (GGM) (Lauritzen, 1996), which estimates an undirected network of partial correlation coefficients (Epskamp et al., 2018). GGM was obtained using a Graphical Least Absolute Shrinkage and Selection Operator (glasso; Friedman et al., 2008, 2014) method, which directly penalizes and shrinks the partial correlation coefficients of the matrix, and thus protects against overfitting. Glasso employs the extended Bayesian information criterion to retrieve model with best fit to the data. The number of factors in the network model is estimated using the walktrap algorithm (Pons and Latapy, 2006), which detects the number and content of distinct subcomponents of uniquely related nodes through the process of “random walks” over the network.

The stability of number of factors and the robustness of each item’s placement within their assigned factors identified using EGA was investigated using bootstrap exploratory graph analysis (bootEGA) (Christensen and Golino, 2021). bootEGA assesses the factorial stability using structural consistency measure, which is the proportion of times each factor initially derived from EGA was replicated from the bootstrap samples. The non-parametric approach of bootEGA was used, which is recommended for skewed data, applies resampling with replacement technique to generate 1,000 samples from the original dataset with the same number of cases. For each generated sample, a network is estimated and walktrap algorithm is employed to create a sampling distribution of networks. Descriptive statistics (median, standard error, 95% confidence intervals) on the number of identified factors, and the proportion of times a specific number of factors was estimated were computed across the replica of networks. Additionally, a median network plot of a sampling distribution of 1,000 networks was generated, which depicts the most likely and stable factor structure identified in the data. The median network plot was compared with the initial EGA plot.

An additional measure to structural consistency is item stability, which calculates a proportion of times each item was present in each factor specified through EGA across the replica of networks. Items identified to replicate in their confirmatory factors ≥80% of the time were considered stable and contributing to structural consistency, while the items with low proportions of replications (< 0.80) were labelled as possibly problematic due to their multidimensional nature and leading to structural inconsistency.

#### Measurement invariance

Measurement invariance investigations were employed to evaluate whether the S-Five can measure the underlying construct of misophonia equivalently across different genders. This procedure ensures that any observed score differences between groups are genuine reflections of actual differences in the construct being measured, rather than being influenced by biases in the measurement tool itself. The measurement invariance of the tool was assessed using multiple indicators, multiple causes model (MIMIC) (Joreskog and Goldberger, 1975; Muthén, 1979), which involves regressing the latent factors and indicators (items) onto a gender covariate. The direct effects of gender on the items were estimated. A significant direct effect signifies measurement-noninvariance (bias) of that item; that is, when the levels of misophonia are held constant, gender alone influences the probability of endorsing a particular item. The analysis was adjusted for age.

#### Reliability

The internal consistency of the S-Five was assessed using Cronbach’s alpha (α; Cronbach, 1951) and McDonald’s omega (ω; McDonald, 1999) within each factor. While α is the most used measure of reliability, Cronbach’s α produces incorrect estimates of internal consistency except in scarce conditions when all factor loadings are of equal magnitude within a scale and no correlated residuals are present (Sijtsma, 2009). Thus, ω coefficient is additionally computed as model-based estimates of internal consistency are favoured (Revelle and Condon, 2019). Guidelines for internal consistency coefficients vary and range from 0.70 to 0.95 (Nunnally and Bernstein, 1994; DeVellis, 2003) but in this study we follow the guidelines outlined by Nunnally (1978) that recommend a value of ≥0.80 for research scales. As a part of internal consistency investigations, additional indices were investigated for each item, namely corrected item-total correlations (ITC), average inter-item correlations (IIC), and alpha/omega if item deleted (AID/OID). For ITC and IIC, the acceptable range of correlation considered was 0.2 to 0.8. Items with values of AID/OID higher than alpha/omega for the total subscale are decreasing the reliability of the scale and were thus labelled as problematic.

The stability of the items and scales across time (test–retest reliability) was assessed using the mixed effects, absolute agreement intraclass correlation coefficient (ICC; Shrout and Fleiss, 1977) and the Psi Non-Parametric Concordance Coefficient (Psi; Kuiper and Hoogenboezem, 2019). Landis and Koch (1977) guidelines were followed for interpreting the results, where evidence towards very good agreement was granted with values >0.81, good agreement was indicated by values ranging between 0.61 to 0.80, moderate agreement was demonstrated for values of 0.41 to 0.60, fair agreement was evidenced with values of 0.21–0.40, and values indicating poor agreement were < 0.20.

#### Validity

The concurrent convergent validity of the S-Five was tested by assessing correlations with pre-existing measures of misophonia, namely MisoQuest and MQ. In comparison, discriminant validity was evaluated through associations with a subscale of MQ (MSYS, sensitivity to specific triggers in comparison to other people), which although related to misophonia, is not measured by the S-Five. To establish convergent validity, the S-Five and its subscales should be at least modestly correlated with MisoQuest and MQ. Discriminant validity should be determined through weak correlations with MSYS subscale of MQ.

All statistical analyses were performed using R version 1.31093 (R Core Team, 2020), M*plus* 8 (Muthén and Muthén, 1998–2012) and SPSS version 28.0 (IBM Corp, 2021) Exploratory factor analysis was run in M*plus* via *MplusAutomation* package (Hallquist and Wiley, 2018) in R and the results of parallel analysis were depicted using *ggplot2* (Wickham, 2016). EGA and bootEGA were conducted using the R package *EGAnet* (Golino et al., 2020), which makes use of the *qgraph* (Epskamp et al., 2012) to visualise the network models. Test–retest reliability analysis was run in the R package *nopaco* (Kuiper and Hoogenboezem, 2019) and internal consistency analysis was conducted in SPSS.

## Results

### Demographic characteristics of the sample

The study included 288 participants from the Polish population (99% were born in Poland), of which 81.3% (*n* = 234) self-identified as having misophonia, 13.1% (*n* = 13) did not and 8.7% (*n* = 25) was not sure (5.6%, *n* = 16 missing). Among the participants, 231 (80.2%) were women, including 2 (0.7%) who identified as trans women; 38 (13.2%) were men, including 17 (6.3%) who identified as trans men; and 19 (6.6%) identified as non-binary/other. The participants’ ages ranged from 18 to 59 years old, with a mean age of 30.15 (standard deviation, SD = 8.39). The characteristics of the participants’ highest level of education was as follows: lower secondary education (*n* = 5, 1.7%), secondary education (*n* = 87, 30.2%), vocational education (*n* = 4, 1.4%), post-secondary non-tertiary education (*n* = 20, 6.9%), Bachelor’s degree (*n* = 66, 22.9%), Master’s degree (*n* = 99, 34.4%), and Doctoral degree (*n* = 7, 2.4%).

The most frequently reported mental health conditions diagnosed by a specialist were depression (*n* = 64, 22.2%) and generalized anxiety disorder (*n* = 44, 15.3%). In terms of audiological diagnoses, 18 (6.3%) participants reported having official diagnoses of tinnitus, 16 (5.6%) of hyperacusis, and 2 (0.7%) of auditory processing disorder.

For test–retest purposes, a subset of 68 participants was re-administered the S-Five three weeks after the initial assessment. This subsample had a mean age of 29 (SD = 7.5), with 56 (82.4%) identifying as women. Missingness in the trigger sections of the S-Five ranged from 2.1 to 4.2%, and listwise deletion was used, resulting in 47 participants for the test–retest analysis of S-Five-T.

### S-Five: statements

#### Dimensionality

**Figure 1:**
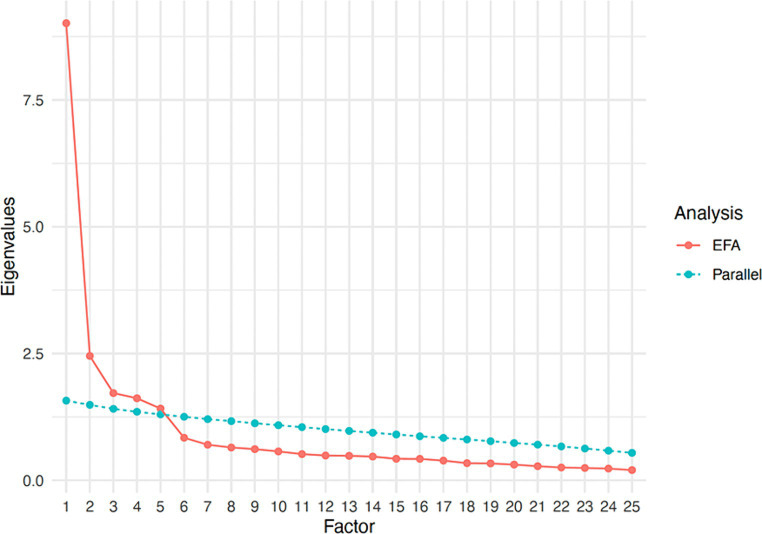
Scree plot showing parallel analysis results.

The data were first evaluated with respect to their suitability for factor analysis, which revealed amenability to factoring (KMO = 0.91; Bartlett’s test χ^2^ = 3748.40, df = 300, *p* < 0.001). Oblimin-rotated EFA was conducted on all 25 items. Five eigenvalues were above 1 (9.014, 2.453, 1.721, 1.619, 1.417) in the sample correlation matrix, suggesting up to five factors to be retained according to the Kaiser-Guttman criterion, explaining the 64.9% of the total variance. Parallel analysis also indicated that the five-factor structure was suitable to the data (see Figure 1). The-five factor structure had close fit to the data [relative χ^2^ = 1.70, RMSEA (90% CI) = 0.05 (0.04, 0.06), CFI = 0.96, TLI = 0.93, SRMR = 0.03]. The factor loading estimates indicated that all items were strongly related to their corresponding factors (λ range = 0.34–0.84; please see Table 1 for loadings). Salient cross-loadings (λ >0.25) were not present for any item. The intercorrelations among the five factors ranged from 0.20 (externalizing-internalizing) to 0.49 (impact-threat). Each factor consisted of five items that conceptually corresponded to the original model identified by Vitoratou et al. (2021b). Solutions with fewer factors did not emerge close fit to the data and increasing the number of factors resulted in factors whose items had non-salient loadings.

**Table 1:** Descriptive indices, associations with age and gender, factor analysis loadings to factors, and reliability indices of the 25 S-Five items (*N* = 288).

S-Five-E statements per factor	Mean (SD)	Median (Q1-Q3)	Mode (min-max)	Age rho	*λ*	Psi (95% CI)	ICC
Externalizing							
I06 Others avoid making noises	7.22 (3.06)	8 (5–10)	10 (0–10)	**0.15	0.73	0.83 (0.79, 1)	0.87
I13 Others should not make sounds	7.24 (3.41)	9 (5–10)	10 (0–10)	0.09	0.65	0.83 (0.79, 1)	0.87
I16 Others selfish	5.73 (3.44)	6 (3–9)	10 (0–10)	0.04	0.66	0.83 (0.79, 1)	0.87
I21 Others bad manners	6.88 (3.15)	8 (5–10)	10 (0–10)	0.04	0.81	0.78 (0.73, 1)	0.85
I25 Others disrespectful	7.59 (2.99)	9 (6–10)	10 (0–10)	0.09	0.71	0.80 (0.76, 1)	0.86
Internalizing							
I05 Respect myself less	3.98 (3.58)	3 (1–7)	0 (0–10)	*-0.13	0.79	0.86 (0.82, 1)	0.88
I08 Unlikeable person	5.69 (3.41)	6 (3–9)	10 (0–10)	−0.11	0.51	0.84 (0.81, 1)	0.87
I12 Angry person inside	6.76 (3.43)	8 (5–10)	10 (0–10)	0.01	0.41	0.84 (0.81, 1)	0.87
I18 Bad person inside	4.69 (3.74)	5 (1–8)	0 (0–10)	**-0.159	0.79	0.82 (0.77, 1)	0.86
I19 Dislike self	7.09 (3.32)	8 (5–10)	10 (0–10)	−0.06	0.75	0.84 (0.80, 1)	0.87
Impact							
I01 Do not meet friends	3.59 (3.21)	3 (1–6)	0 (0–10)	0.04	0.63	0.85 (0.82, 1)	0.88
I09 Eventually isolated	4.59 (3.37)	5 (2–8)	0 (0–10)	−0.09	0.64	0.85 (0.81, 1)	0.87
I14 Avoid places	5.17 (3.61)	5 (2–9)	10 (0–10)	0.07	0.71	0.83 (0.79, 1)	0.87
I15 Cannot do everyday things	4.17 (3.6)	3 (1–7)	0 (0–10)	−0.08	0.84	0.82 (0.77, 1)	0.86
I20 Limited job opportunities	3.45 (3.41)	2 (0–6)	0 (0–10)	−0.08	0.68	0.82 (0.79, 1)	0.86
Outburst							
I04 Verbally aggressive	7.32 (2.85)	8 (5–10)	10 (0–10)	−0.02	0.48	0.81 (0.77, 1)	0.86
I17 Physically aggressive	4.23 (3.63)	4 (1–7)	0 (0–10)	0.06	0.75	0.82 (0.78, 1)	0.86
I22 Violence	3.07 (3.22)	2 (0–5)	0 (0–10)	−0.08	0.60	0.82 (0.78, 1)	0.86
I23 Shout at people	6.42 (3.62)	8 (3–10)	10 (0–10)	−0.1	0.67	0.82 (0.79, 1)	0.87
I24 Afraid of outburst	7.34 (2.97)	8 (6–10)	10 (0–10)	−0.01	0.34	0.80 (0.76, 1)	0.86
Threat							
I02 Panic or explode	8.16 (2.5)	9 (7–10)	10 (0–10)	−0.12^*^	0.52	0.82 (0.78, 1)	0.86
I03 Feel helpless	8.48 (2.53)	10 (8–10)	10 (0–10)	−0.05	0.60	0.76 (0.72, 1)	0.84
I07 Feel anxious	8.55 (2.36)	10 (8–10)	10 (0–10)	0.04	0.82	0.77 (0.73, 1)	0.85
I10 Experience distress	7.92 (3.01)	10 (7–10)	10 (0–10)	−0.08	0.71	0.82 (0.78, 1)	0.86
I11 Feel trapped	8.76 (2.11)	10 (8–10)	10 (0–10)	−0.02	0.80	0.78 (0.74, 1)	0.85

SD, standard deviation; Q1-Q3, first and third quartile; rho: Spearman’s correlation coefficient; λ, EFA loadings; Psi coefficient and 95% confidence intervals; ICC, intraclass correlation coefficient (two-way mixed effects, absolute agreement); *p < 0.05; ^**^p < 0.01. All EFA loadings are significant, *p* < 0.001.

**Figure 2:**
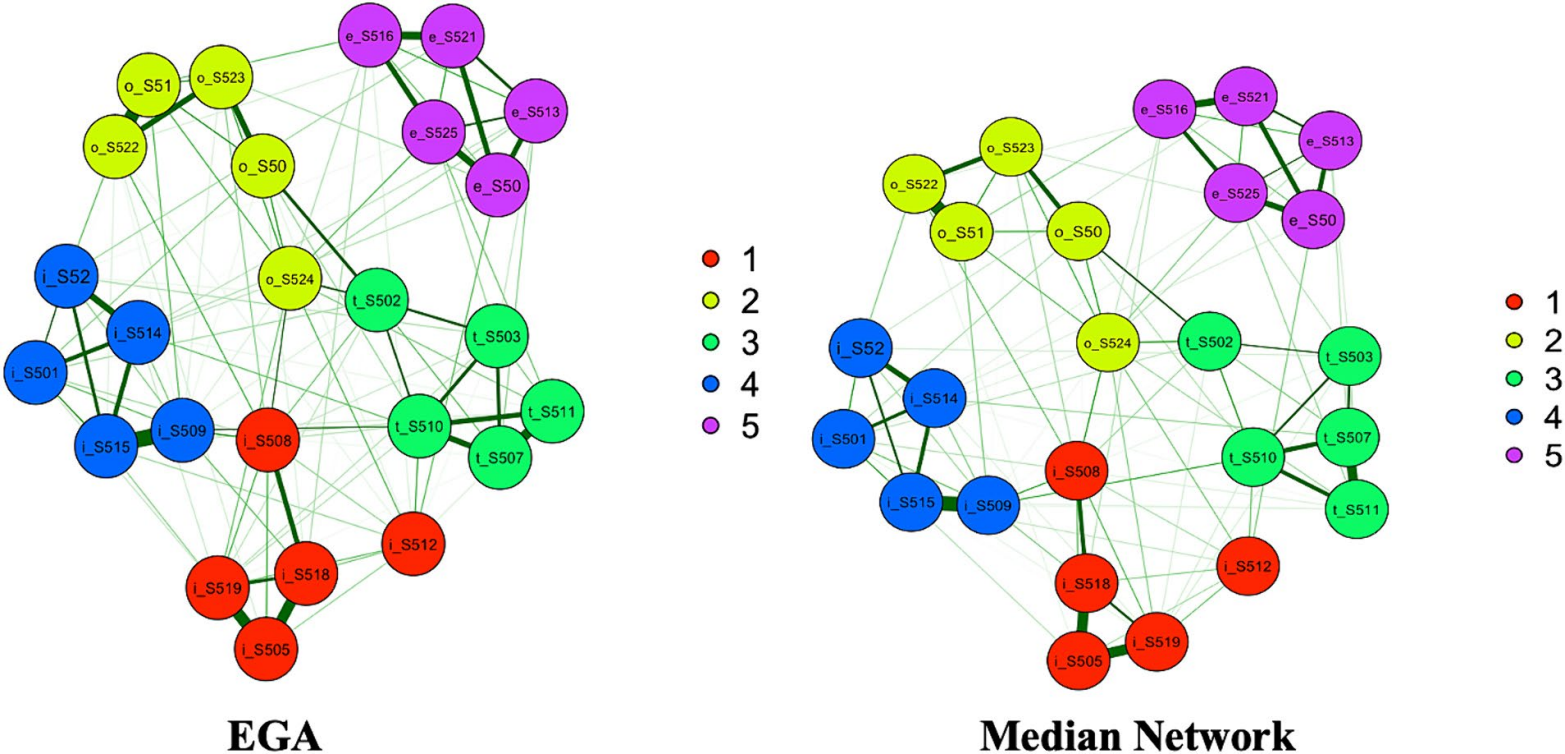
EGA (left) and bootstrap EGA’s median network (right) for the S-Five model. Dimension 1 (red) relates to internalizing appraisals, dimension 2 (light green) relates to outburst, dimension 3 (dark green) corresponds to threat, dimension 4 (blue) relates to impact on life, and dimension 5 (violet) indicates externalizing appraisals.

The dimensionality of the S-Five was further investigated using EGA, which also concluded to 5 dimensions (Figure 2) whose items correspond to the theoretical factors identified by Vitoratou et al. (2021b).

The stability and reproducibility of this factor structure was evaluated using the dimension stability function of bootstrap EGA (bootEGA) for 1,000 iterations. The median network structure (Figure 2) consisted of 5 dimensions and was identical to the empirical EGA. 5-factor structure was stable across 1,000 bootstrapped samples (median = 5, SE = 0.29, and 95% CI [4.43, 5.57]). The frequency of factorial solutions derived from bootEGA across all replica of networks was computed, which showed that the five factor structure was replicated the most (919 times out of 1,000 samples). While bootEGA identified 4, 6, and 7 factors to be present across all 1,000 bootstrap replicate samples, these were only replicated 25, 56, and 1 time, respectively. Further stability checks were conducted by assessing stability of each dimension individually to see how frequently each of the initial EGA factors (identical item allocation) is reproduced across replica of networks. Dimensions 4 and 5 were the most stable, with 94 and 100% proportion of times when identical item arrangement was replicated, respectively. Dimensions 1, 2, and 3 were less stable and were reproduced 59, 62 and 66% times, respectively.

**Figure 3:**
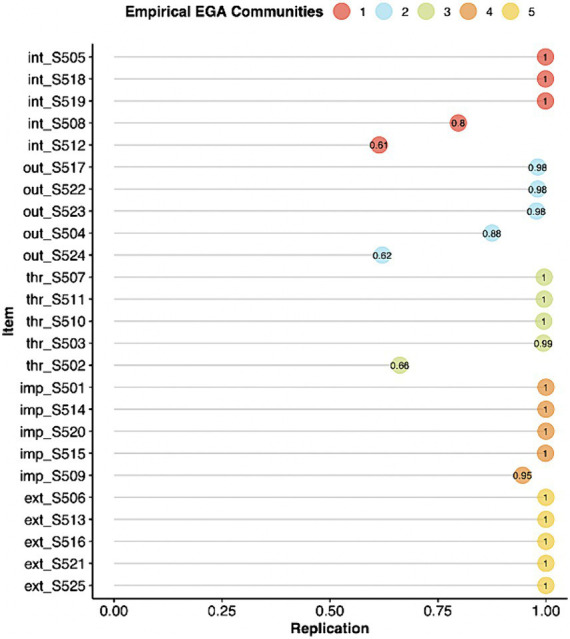
Proportion of times each item was replicated in their designated dimensions across 1,000 bootstrapped samples. 1 (red) signifies internalizing appraisals, 2 (blue) corresponds to outburst, 3 (green) indicates threat, 4 (orange) refers to impact on life, and 5 (yellow) signifies externalizing appraisals.

Items were assessed for their stability of placement in each designated factor in the bootstrapped samples (please see Figure 3). All but three items (thr_S502, out_S524, int_S512) had high proportion of times (≥ 0.80) they were replicated in 1,000 bootstrap samples in the confirmatory dimensions, indicating that these items are stable and consistently identified in their theoretical factors. Items thr_S502, out_S524, and int_S512 had relatively low proportions (< 0.80) of replications, indicating that these items could be considered unstable. These unstable items were further investigated to evaluate which additional dimensions they were replicating on (please see Supplementary Table S3 for item stability values for all items). Item int_S512 from the Internalizing dimension was replicated in its corresponding dimension in 614 out of 1,000 bootstrapped samples, but also 213 times on Outburst factor and 122 times on Threat factor. With respect to out_S524, it replicated on its theoretical dimension in 621 samples and 198 times on Internalizing and 128 times on Threat factors. Item from the Threat factor (thr_S502) replicated on its own confirmatory dimension 662 times. However, it was also replicated in the Internalizing dimension (91 times) and Outburst (219 times). Although these items do correspond to their theoretical factors, they overlap conceptually with other factors, which is an indication of their complexity.

#### Measurement invariance

A MIMIC model was fitted to evaluate whether the probability of endorsing an item is influenced by gender adjusted for age. Significant directs effects of gender were found on 4 items, namely imp_S501 (“Do not meet friends”), imp_S514 (“Avoid places”), out_S523 (“Shout at people”), and thr_S510 (“Experience distress”). At any given value of misophonia, women scored 1.08 and 0.95 units higher on out_S523 and thr_S510, respectively, (on a scale of 0 to 10) than men. Men were more likely to endorse imp_S501 and imp_S514, that is, they scored 1.27 and 1.33 units higher, respectively, than women while controlling for the level of misophonia. The remaining 21 items of the S-Five were measurement invariant, that is non-biased with respect to gender (adjusted for age). The direct effects of gender on these four items were negligible, thus allowing comparison of the S-Five scores between women and men. Women scored significantly higher on the S-Five total score and its three subscales (internalizing, threat, outburst) compared to men (see Table 2).

**Table 2:** Norms and internal consistency reliability of the five factors of the S-Five and total score (*N* = 288).

	Mean (SD)	Median (Q1-Q3)	Mode (min-max)	Gender difference	Age rho	Internal consistency			
						ITC	AID	OID	α/ ω
Externalizing	34.65 (12.81)	38 (27–45)	50 (0–50)	*U* = 3,670	0.11	0.57–0.65	0.78–0.80	0.78–0.80	0.86/0.82
Internalizing	28.22 (13.74)	29 (19–40)	32 (0–50)	*U* = 3,102^**^	−0.13^*^	0.64–0.77	0.84–0.87	0.84–0.87	0.87/0.91
Impact	20.98 (13.87)	20 (10–32)	2 (0–50)	*U* = 3,786	−0.04	0.66–0.83	0.88–0.91	0.88–0.91	0.85/0.88
Outburst	28.38 (12.36)	29 (20–38)	27 (0–50)	t (45.339) = 2.829^**^	−0.06	0.75–0.84	0.90–0.91	0.90–0.92	0.81/0.91
Threat	41.88 (10.08)	46 (39–50)	50 (0–50)	*U* = 2855.5^**^	−0.10	0.72–0.84	0.88–0.90	0.88–0.91	0.86/0.92
Total	154.1 (47.29)	160 (129–187)	133 (6–250)	t (267) = 3.416^**^	−0.07	0.44–0.68	0.92–0.92	0.91–0.92	0.92/0.92

SD, standard deviation; Q1-Q3, first and third quartile; U, Mann–Whitney test; t, independent samples *t* test; ITC, item-total correlations; AID, alpha if item deleted; OID, omega if item deleted; ω, McDonald’s omega; α, Cronbach’s alpha; ^*^p < 0.05; ^**^p < 0.01.

#### Reliability

Test–retest reliability at item level indicated very good agreement for both Psi and ICC (Table 1). Internal consistency within each subscale was satisfactory according to McDonald’s omega (ω = 0.82–0.92) and Cronbach’s alpha (α = 0.81–0.87). Further internal consistency investigations with respect to average IIC, ITC, AID, and OID revealed all items to exhibit values within acceptable range (Table 2).

#### Validity

Moderate to strong correlations were found between the S-Five and its subscales and MisoQuest, indicating concurrent convergent validity of the scale (Table 3). Additionally, evidence of concurrent convergent validity was supported by moderate to strong correlations between the S-Five and MQ, as well as its MSES and MEBS subscales. On the other hand, weak correlations were observed between four S-Five factors (except for impact) and MSYS subscale, suggesting distinctiveness of the misophonia aspects measured by these scales. Impact had moderate correlation with MSYS.

**Table 3:** Intercorrelations of the S-Five scores with concurrent validity measures (Spearman’s rho).

	Externalizing	Internalizing	Impact	Threat	Outburst	Total
S-Five (*N* = 288)						
Externalizing	1					
Internalizing	0.26^**^	1				
Impact	0.34^**^	0.50^**^	1			
Threat	0.31^**^	0.52^**^	0.53^**^	1		
Outburst	0.38^**^	0.48^**^	0.49^**^	0.44^**^	1	
Total	0.61^**^	0.75^**^	0.80^**^	0.69^**^	0.74^**^	1
MisoQuest (*N* = 252)						
Total	0.36^**^	0.53^**^	0.69^**^	0.72^**^	0.47^**^	0.70^**^
MQ (*N* = 221)						
MEBS	0.38^**^	0.51^**^	0.63^**^	0.64^**^	0.63^**^	0.71^**^
MSYS	0.26^**^	0.18^**^	0.32^**^	0.26^**^	0.24^**^	0.30^**^
MSES	0.37^**^	0.40^**^	0.64^**^	0.50^**^	0.49^**^	0.65^**^
Total	0.38^**^	0.44^**^	0.59^**^	0.56^**^	0.53^**^	0.63^**^
PHQ-9 (*N* = 264)						
Total	0.04	0.31^**^	0.26^**^	0.22^**^	0.19^**^	0.28^**^
*GAD-7*						
Total	0.15*	0.33^**^	0.25^**^	0.29^**^	0.27^**^	0.35^**^
ASI-3 (*N* = 234)						
Social	−0.02	0.33^**^	0.20^**^	0.15^*^	0.09	0.22^**^
Cognitive	0.08	0.38^**^	0.18^**^	0.19^**^	0.20^**^	0.29^**^
Physical	0.08	0.15^*^	0.09	0.11	0.12	0.14^*^
Total	0.06	0.33^**^	0.18^**^	0.18^**^	0.15^*^	0.25^**^

MQ, Misophonia Questionnaire; PHQ-9, Physical Health Questionnaire; GAD-7, Generalized Anxiety Disorder Assessment; ASI, Anxiety Sensitivity Index; ω, McDonald’s omega. ^*^p < 0.05, ^**^p < 0.001.

The S-Five subscales, with the exception of externalizing, displayed weak to moderate correlations with depression. Notably, the externalizing factor did not exhibit any significant associations with depression. A moderate correlation was observed between S-Five total and its internalizing subscale with anxiety as assessed by GAD-7. In contrast, the remaining subscales were weakly associated with anxiety, particularly the externalizing factor. Furthermore, anxiety sensitivity (ASI-3) and its social and cognitive factors showed moderate correlations with the internalizing factor of S-Five, while very weak correlation was observed with the physical factor. As for the other S-Five factors, ASI-3 and its social and cognitive factors exhibited very weak associations with them excluding the externalizing factor, which exhibited no correlations with any of the ASI-3 subscales. Total S-Five had very weak correlation with physical subscale of ASI-3.

With respect to reactions to triggers, none of the S-Five subscales correlated with irritation, negative and physiological reactions (Table 4). Externalizing factor only correlated with anger. The remaining factors had associations with distress, anger, panic and were negatively related to no feeling. Outburst factor was additionally negatively associated with disgust and positive reactions. The total S-Five score emerged positively correlated with distress, anger and panic, and negatively correlated with no feeling and disgust.

**Table 4:** Intercorrelations of the S-Five-T and correlations with other measures (Spearman’s rho).

	No feeling	Irritation	Distress	Disgust	Anger	Panic	Negative	Positive	Physiological	TC	FIRS	RIRS
S-Five RC (*N* = 222)												
Irritation	−0.42^**^											
Distress	−0.26^**^	−0.04										
Disgust	−0.16^*^	0.07	0.01									
Anger	−0.37^**^	−0.11	0.03	−0.22^**^								
Panic	−0.27^**^	−0.19^**^	0.18^**^	0.12	−0.07							
Negative	−0.17^*^	−0.08	−0.07	0.08	−0.23^**^	0.1						
Positive	0.02	−0.15^*^	0.05	0.03	−0.16*	0.1	0.1					
Physiological	−0.05	−0.09	0.05	0.05	−0.14^*^	0	0.25^**^	0.16^*^				
TC (*N* = 219)	−0.99^**^	0.43^**^	0.24^**^	0.15^*^	0.38^**^	0.25^**^	0.17^*^	−0.14^*^	0.04			
FIRS (*N* = 219)	−0.82^**^	0.24^**^	0.24^**^	0.03	0.48^**^	0.28^**^	0.06	−0.12	−0.03	0.83^**^		
RIRS (*N* = 219)	−0.33^**^	−0.10	0.13	−0.11	0.40^**^	0.21^**^	−0.13^*^	−0.07	−0.10	0.34^**^	0.76^**^	
S-Five (*N* = 222)												
Externalizing	−0.10	−0.02	0.05	−0.08	0.15^*^	−0.05	0.03	−0.1	0.02	0.12	0.27^**^	0.36^**^
Internalizing	−0.24^**^	−0.02	0.17^**^	−0.10	0.22^**^	0.16^*^	−0.05	−0.06	−0.06	0.25^**^	0.31^**^	0.26^**^
Impact	−0.28^**^	−0.09	0.19^**^	−0.10	0.23^**^	0.30^**^	−0.01	−0.03	0.04	0.28^**^	0.40^**^	0.40^**^
Outburst	−0.22^**^	−0.08	0.14^*^	−0.17**	0.27^**^	0.15^*^	−0.04	−0.15*	0.03	0.24^**^	0.38^**^	0.42^**^
Threat	−0.26^**^	−0.13	0.25^**^	−0.06	0.17^**^	0.29^**^	−0.04	−0.05	0.02	0.28^**^	0.43^**^	0.45^**^
Total	−0.26^**^	−0.10	0.20^**^	−0.14*	0.24^**^	0.22^**^	−0.01	−0.1	0.03	0.28^**^	0.45^**^	0.47^**^
MisoQuest (*N* = 208)												
Total	−0.24^**^	−0.12	0.24^**^	−0.14*	0.30^**^	0.24^**^	−0.1	−0.03	0.02	0.26^**^	0.43^**^	0.48^**^
MQ (*N* = 210)												
MEBS (*N* = 183)	−0.21^**^	−0.13	0.20^**^	−0.17*	0.25^**^	0.28^**^	−0.08	−0.14	−0.01	0.22^**^	0.41^**^	0.46^**^
MSYS (*N* = 183)	−0.73^**^	0.30^**^	0.18^**^	0.08	0.37^**^	0.22^**^	0.11	−0.13	−0.001	0.75^**^	0.77^**^	0.45^**^
MSES (*N* = 210)	−0.27^**^	−0.06	0.18^**^	−0.12	0.30^**^	0.20^**^	−0.03	−0.08	0.06	0.29^**^	0.46^**^	0.46^**^
Total (*N* = 183)	−0.53^**^	0.07	0.26^**^	−0.09	0.37^**^	0.32^**^	0.02	−0.16*	−0.006	0.56^**^	0.70^**^	0.56^**^
PHQ-9 (*N* = 215)												
Total	−0.25^**^	−0.01	0.27^**^	0.12	0.1	0.20^**^	−0.01	0.02	0.02	0.26^**^	0.26^**^	0.14*
GAD-7 (*N* = 217)												
Total	−0.22^**^	−0.05	0.20^**^	0.08	0.12	0.15^*^	−0.02	−0.08	−0.02	0.24^**^	0.29^**^	0.23^**^
ASI-3 (*N* = 195)												
Social	−0.24^**^	0.08	0.07	0.26^**^	0.07	0.16^*^	−0.05	−0.01	0	0.25^**^	0.18^*^	0.06
Cognitive	−0.27^**^	0.1	0.1	0.18^*^	0.05	0.17^*^	−0.06	0	−0.05	0.27^**^	0.21^**^	0.08
Physical	−0.27^**^	0.11	0.06	0.16^*^	0.14	0.08	−0.07	−0.03	−0.06	0.27^**^	0.23^**^	0.14
Total	−0.29^**^	0.12	0.09	0.24^**^	0.09	0.17^*^	−0.08	−0.02	−0.04	0.30^**^	0.23^**^	0.10

RC, reaction count; TC, trigger count; FIRS, Frequency-intensity reaction score; RIRS, relative intensity reaction score; MQ, Misophonia Questionnaire; PHQ-9, Physical Health Questionnaire; GAD-7, Generalised Anxiety Disorder Assessment; ASI-3, Anxiety Sensitivity Index. ω, McDonald’s omega. ^*^p < 0.05, ^**^p < 0.001.

### S-Five: triggers

#### Stability

With respect to test–retest reliability of the reaction counts (RC), all RCs demonstrated good to very good agreement ranging from 0.74 (physiological reaction) to 0.91 (no feeling) with respect to Psi (Table 5). ICC values ranged from 0.84 (physiological reaction) to 0.91 (no feeling), also indicating very good agreement in time. Psi coefficients for RIRS, FIRS and TC were very good (>0.86) and ICC also indicated very good agreement (>0.88). With respect to the intensity trigger sounds, Psi ranged from 0.74 (footsteps) to 0.91 (muffled sounds, baby crying) indicating good to very good agreement, ICC values showed very good agreement (0.83–0.90) for all sounds.

**Table 5:** Norms and reliability of the S-Five-T scores.

	Mean (SD)	Median (Q1-Q3)	Mode (min-max)	Gender difference (U)	Age rho	Stability (*N* = 36)	
						Psi (95% CI)	ICC
No feeling (*N* = 222)	12.36 (6.19)	12 (8–16)	11 (0–33)	3341.5^**^	−0.03	0.91 (0.87, 1)	0.91
Irritation (*N* = 222)	10.65 (4.47)	10 (8–13)	9 (1–23)	2076	−0.01	0.82 (0.75, 1)	0.86
Distress (*N* = 222)	1.02 (1.81)	0 (0–1)	0 (0–11)	1982.5	−0.09	0.78 (0.73, 1)	0.85
Disgust (*N* = 222)	2.18 (2.18)	2 (0–3)	0 (0–9)	2329.5	−0.08	0.85 (0.81, 1)	0.88
Anger (*N* = 222)	6.54 (4.66)	6 (3–9)	6 (0–23)	2081.5	0.11	0.85 (0.79, 1)	0.87
Panic (*N* = 222)	1.55 (2.86)	0 (0–2)	0 (0–20)	2,249	−0.12	0.83 (0.78, 1)	0.87
Negative (*N* = 222)	1.99 (2.41)	1 (0–3)	0 (0–14)	1756^*^	0.02	0.79 (0.74, 1)	0.85
Positive (*N* = 222)	0.38 (0.87)	0 (0–0)	0 (0–5)	2778.5	−0.16^*^	0.78 (0.72, 1)	0.85
Physiological (*N* = 222)	0.33 (1.18)	0 (0–0)	0 (0–11)	2,247	0.04	0.74 (0.7, 1)	0.84
TC (*N* = 221)	24.29 (6.25)	25 (21–28)	26 (4–37)	1,360^**^	0.04	0.91 (0.87, 1)	0.91
FIRS (*N* = 221)	162.22 (64.14)	157 (120–201)	133 (12–342)	1,331^**^	0.13*	0.93 (0.88, 1)	0.91
RIRS (*N* = 221)	6.54 (1.50)	6.58 (5.65–7.50)	5.84 (0.75–9.50)	1827.5	0.20*	0.86 (0.8, 1)	0.88

SD, standard deviation; Q1-Q3, first and third quartile; U, Mann–Whitney test; rho, Spearman’s correlation coefficient; Psi coefficient and 95% confidence intervals; ICC, intraclass correlation coefficient (two-way mixed effects, absolute agreement); ^*^p < 0.05; ^**^p < 0.01.

#### Reaction counts

No feeling was on average the most frequently reported reaction. Specifically, an average of 12 out of 37 trigger sounds did not evoke any reactions, followed by irritation (10 out of 37) and anger (6 out of 37). With respect to differences in scores, women scored significantly higher on the negative RC than men whilst men had significantly higher score on no feeling RC.

Distress, anger, panic emerged weakly correlated with MisoQuest and MQ and all its subscales. Additionally, MQ had very weak negative correlations with positive reaction. MSYS was also moderately associated with irritation, whereas MEBS emerged negatively and very weakly correlated with disgust. The measures of depression and anxiety were only weakly correlated with distress and panic. All subscales of ASI-3 had a weak association with disgust and all but physical subscale had weak correlations with panic.

All S-Five subscales were weakly to moderately correlated with FIRS and RIRS and all with the exception of externalizing subscale correlated weakly with TC. MisoQuest and MQ, the other measures of misophonia, were weakly to strongly associated with FIRS, RIRS, and TC. Depression and anxiety were weakly correlated with TC and FIRS, while anxiety only showed a very weak association with RIRS. TC and FIRS had weak correlations with all ASI-3 subscales, while RIRS had no significant correlations with any ASI-3 subscales.

#### Intensity

**Figure 4:**
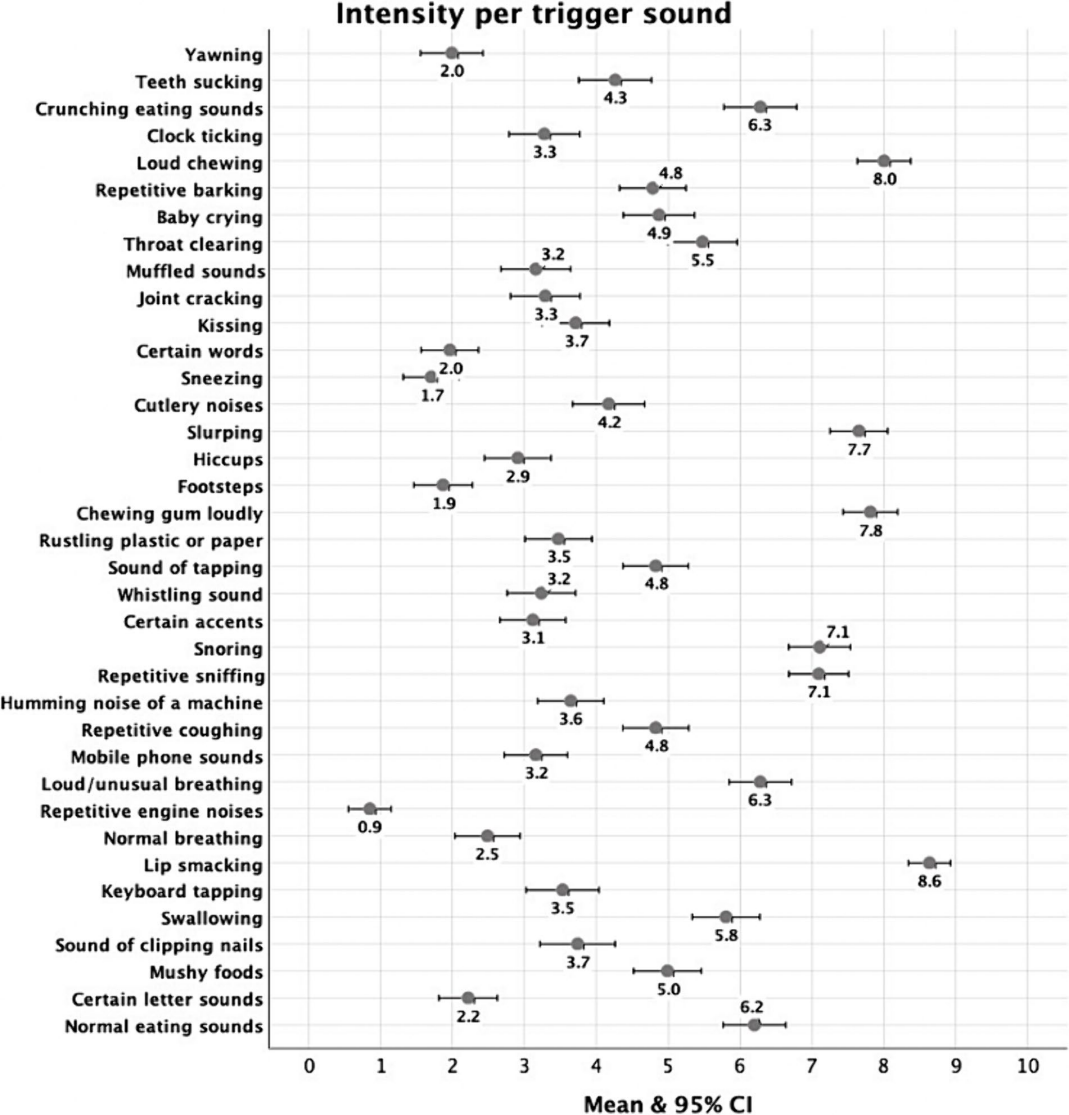
Mean intensity for each selected trigger.

Descriptive indices for intensity of sounds are presented in Table 6 and mean intensity of each trigger sound is represented in Figure 4. The sounds for which the highest intensity of reactions was evoked include lip smacking, loud chewing, chewing gum, slurping, snoring, loud/unusual breathing, crunching, and normal eating sounds. The lowest intensity was reported for yawning, certain words, footsteps, sneezing, and repetitive engine noises. Women had significantly higher intensity of reactions on sound of clipping nails, swallowing, lip smacking, normal breathing, loud unusual breathing, repetitive coughing, repetitive sniffing, snoring, chewing gum, slurping, muffled sounds, throat clearing, clock ticking, crunching, teeth sucking.

**Table 6:** Norms and reliability of the intensity items for the 37 S-Five-T sounds.

Trigger sounds	Mean (SD)	Median (Q1-Q3)	Mode (min-max)	Gender difference (U)	Age rho	Stability (*N* = 36)	
						Psi (95% CI)	ICC
Normal eating sounds (*N* = 279)	6.25 (3.27)	7 (4–9)	8 (0–10)	3536.5	−0.02	0.88 (0.83, 1)	0.89
Certain letter sounds (*N* = 258)	2.18 (3.02)	0 (0–5)	0 (0–10)	3406.5	−0.06	0.81 (0.76, 1)	0.86
Mushy foods (*N* = 253)	5.09 (3.56)	6 (1–8)	0 (0–10)	2,977	−0.05	0.83 (0.77, 1)	0.87
Sound of clipping nails (*N* = 251)	3.64 (3.9)	3 (0–7)	0 (0–10)	2233^*^	0.04	0.85 (0.8, 1)	0.88
Swallowing (*N* = 251)	5.77 (3.61)	6 (3–9)	10 (0–10)	2241^*^	0.05	0.89 (0.84, 1)	0.89
Keyboard tapping (*N* = 250)	3.53 (3.76)	3 (0–7)	0 (0–10)	2709.5	0.01	0.84 (0.79, 1)	0.87
Lip smacking (*N* = 249)	8.62 (2.26)	10 (8–10)	10 (0–10)	2426.5^*^	0.07	0.81 (0.74, 1)	0.86
Normal breathing (*N* = 248)	2.53 (3.45)	0 (0–5)	0 (0–10)	2313.5*	− 0.12^*^	0.82 (0.77, 1)	0.86
Repetitive engine noises (*N* = 248)	0.87 (2.28)	0 (0–0)	0 (0–10)	2904.5	−0.12	0.75 (0.7, 1)	0.84
Loud/unusual breathing (*N* = 245)	6.32 (3.21)	7 (4–9)	10 (0–10)	2102^*^	0.00	0.87 (0.82, 1)	0.88
Mobile phone sounds (*N* = 245)	3.11 (3.31)	2 (0–6)	0 (0–10)	2810.5	−0.01	0.85 (0.79, 1)	0.88
Repetitive coughing (*N* = 243)	4.95 (3.41)	5 (2–8)	0 (0–10)	2092^*^	0.07	0.86 (0.82, 1)	0.88
Humming (*N* = 242)	3.64 (3.48)	3 (0–7)	0 (0–10)	2,303	0.19^**^	0.83 (0.78, 1)	0.87
Repetitive sniffing (*N* = 242)	7.2 (3.04)	8 (5–10)	10 (0–10)	1,691^**^	0.20^**^	0.87 (0.82, 1)	0.88
Snoring (*N* = 241)	7.15 (3.19)	8 (5–10)	10 (0–10)	2021.5^*^	0.15^**^	0.9 (0.87, 1)	0.9
Certain accents (*N* = 241)	3.13 (3.42)	2 (0–6)	0 (0–10)	2,403	0.13^*^	0.79 (0.73, 1)	0.85
Whistling sound (*N* = 241)	3.12 (3.54)	2 (0–6)	0 (0–10)	2,405	0.21^**^	0.83 (0.76, 1)	0.87
Sound of tapping (*N* = 238)	4.73 (3.42)	5 (1–7)	0 (0–10)	2,256	0.27^**^	0.84 (0.79, 1)	0.87
Rustling plastic or paper (*N* = 238)	3.38 (3.47)	3 (0–6)	0 (0–10)	2420.5	0.20^**^	0.81 (0.75, 1)	0.86
Chewing gum (*N* = 237)	7.72 (2.93)	9 (6–10)	10 (0–10)	*2108	0.16^**^	0.84 (0.8, 1)	0.87
Footsteps (*N* = 236)	1.79 (3.02)	0 (0–3)	0 (0–10)	2438.5	−0.08	0.74 (0.69, 1)	0.83
Hiccups (*N* = 236)	2.92 (3.43)	2 (0–6)	0 (0–10)	2,384	0.02	0.84 (0.79, 1)	0.87
Slurping (*N* = 234)	7.66 (3.01)	9 (6–10)	10 (0–10)	1917.5^*^	0.18^**^	0.87 (0.82, 1)	0.88
Cutlery noises (*N* = 233)	4.14 (3.77)	4 (0–8)	0 (0–10)	2,491	0.08	0.86 (0.81, 1)	0.88
Sneezing (*N* = 233)	1.72 (2.92)	0 (0–3)	0 (0–10)	2277.5	0.04	0.77 (0.72, 1)	0.85
Certain words (*N* = 233)	1.97 (2.99)	0 (0–4)	0 (0–10)	2,177	−0.03	0.81 (0.75, 1)	0.86
Kissing (*N* = 232)	3.6 (3.48)	3 (0–7)	0 (0–10)	2,192	0.02	0.8 (0.74, 1)	0.86
Joint cracking (*N* = 233)	3.32 (3.61)	2 (0–7)	0 (0–10)	2,345	0.14^*^	0.86 (0.82, 1)	0.88
Muffled sounds (*N* = 232)	3.06 (3.61)	1 (0–6)	0 (0–10)	1887.5^*^	−0.02	0.91 (0.87, 1)	0.9
Throat clearing (*N* = 229)	5.5 (3.61)	6 (2–9)	0 (0–10)	1552^*^	0.15^*^	0.84 (0.79, 1)	0.87
Baby crying (*N* = 228)	4.82 (3.75)	5 (0–8)	0 (0–10)	2769.5	−0.11	0.91 (0.87, 1)	0.9
Repetitive barking (*N* = 227)	4.74 (3.46)	5 (2–8)	0 (0–10)	2081	0.11	0.84 (0.78, 1)	0.87
Loud chewing (*N* = 227)	7.96 (2.8)	9 (7–10)	10 (0–10)	1921.5	0.02	0.83 (0.78, 1)	0.87
Clock ticking (*N* = 225)	3.24 (3.69)	2 (0–7)	0 (0–10)	1680^*^	0.13^*^	0.85 (0.8, 1)	0.88
Crunching (*N* = 224)	6.27 (3.81)	8 (3–10)	10 (0–10)	1741.5^*^	0.12	0.85 (0.8, 1)	0.88
Teeth sucking (*N* = 224)	4.26 (3.8)	4 (0–8)	0 (0–10)	1792^*^	0.28^**^	0.81 (0.75, 1)	0.86
Yawning (*N* = 224)	1.98 (3.26)	0 (0–3)	0 (0–10)	1978.5	0.14^*^	0.78 (0.73, 1)	0.85

SD: standard deviation; Q1-Q3: first and third quartile; U: Mann–Whitney test; rho: Spearman’s correlation coefficient; Psi coefficient and 95% confidence intervals; ICC: intraclass correlation coefficient (two-way mixed effects, absolute agreement); *p < 0.05; **p < 0.01.

**Figure 5:**
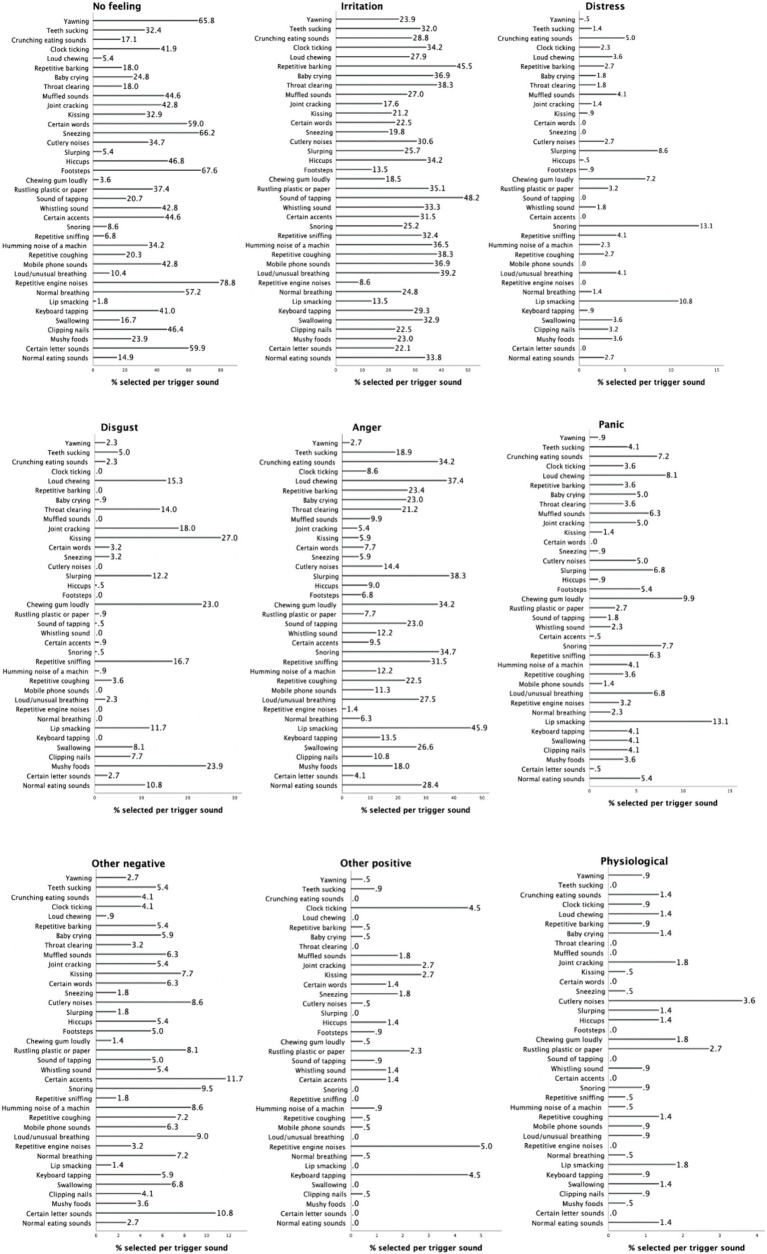
Percentages for each selected trigger per emotional reaction.

Figure 5 illustrates the percentages of participants selecting each of nine emotional reactions to 37 triggers. “Repetitive engine noises” (78.8%), ‘footsteps’ (67.6%), and “sneezing” (66.2%) were selected the most frequently as eliciting no feeling. On the other hand, triggers such as sound of “tapping” (48.2%), “repetitive barking” (45.5%), and “loud/unusual breathing” (39.2%) predominantly evoked irritation. “‘Snoring” (13.1%), “lip smacking” (10.8%) and “slurping” (8.6%) were selected most frequently as causing distress, while sounds such as “kissing” (27%), “mushy foods” (23.9%), and “chewing gum loudly” (23%) most often elicited disgust. The triggers that predominantly induced anger were “lip smacking” (45.9%), “slurping” (38.3%), and “loud chewing” (37.4%). For panic, “lip smacking” (13.1%), “chewing gum loudly” (9.9%), “loud chewing” (8.1%) were selected most frequently. “Certain accents” (11.7%), “certain letter sounds” (10.8%), “snoring” (9.5%) were reported the most frequently to cause other negative emotions whilst “repetitive engine noises” (5%), “keyboard tapping” (4.5%), “clock ticking” (4.5%) were selected most often as eliciting other positive emotions. Finally, physiological reactions were evoked most frequently by “cutlery noises” (3.6%) and “rustling plastic or paper” (2.7%).

## Discussion

The main objective of this study was to translate and validate the Polish version of the S-Five, a comprehensive questionnaire for assessing misophonia symptoms’ severity. The results provided preliminary support for the five-factor structure of the S-Five in the Polish-speaking population, consisting of both individuals with and without self-identified misophonia (not implying a formal diagnosis but potentially suggestive of misophonia), consistent with studies in English-speaking (Vitoratou et al., 2021b), German (Remmert et al., 2022), Chinese (Vitoratou et al., 2022), and Portuguese (Hayes et al., 2024) populations. The factor structure’s robustness was supported through the satisfactory stability of each subscale of the S-Five, that is, they are very likely to reproduce in other samples. Additionally, the questionnaire exhibited strong psychometric properties, including reliability, internal consistency, gender-related measurement invariance, and validity, replicating findings from previous research. The secondary goal of the study was to discuss the differences and similarities of misophonic experiences between the Polish sample and similar studies conducted in other cultural groups.

### Structural validity of the S-Five in a Polish sample

This study provides initial evidence supporting the five theoretical factors (externalizing appraisals, internalizing appraisals, threat, impact, and outburst) of the S-Five questionnaire in the Polish population, highlighting the cross-cultural validity of using the S-Five to measure misophonia. To investigate the factor structure more thoroughly, a novel method called bootEGA was employed to examine the stability of these misophonic dimensions and their item allocations across 1,000 simulated samples (known as bootstrapped samples) from the original dataset. The five-factor structure was replicated with a high frequency (92% of the time), making the dimensions of the S-Five generalizable to other samples. This was further supported by successful replications of the five factors in representative sample of the UK population (Vitoratou et al., 2023), German (Remmert et al., 2022), Mandarin-speaking Chinese (Vitoratou et al., 2022), and Portuguese (Hayes et al., 2024) populations.

Among the five aspects of misophonic experience assessed by the S-Five questionnaire, externalizing appraisals and impact factors demonstrated the highest structural consistency. This means that the items belonging to these factors consistently grouped together in the same dimensions. Specifically, all externalizing items were allocated to their factor 100% of the time. The remaining factors were also stable, but to a lesser extent, with the internalizing factor showing the lowest structural consistency.

The stability issues with the internalizing dimension were mainly due to one specific item: ‘I feel like I must be a very angry person inside because of the way I react to certain sounds.’ This item shared some conceptual similarity with the outburst factor, and at times, it replicated on the outburst factor instead of its intended internalizing factor. The item’s focus on feelings of anger, rather than lack of respect or self-dislike, possibly explained its overlap with the outburst factor. The exploratory factor analysis also reflected this finding, as the item had the weakest association with its main factor compared to the other internalizing items. This result also aligns with the findings from the Mandarin version, where this item emerged with a cross-loading (Vitoratou et al., 2022).

Regarding the outburst factor, one item—'I am afraid I will do something aggressive or violent because I cannot stand the noise someone is making’—occasionally replicated on both the internalizing and threat dimensions. The first part of the item suggested a fear of emotions escalating, characteristic of the threat factor, while its association with the internalizing factor indicated worries about potential outbursts, leading to intrusive thoughts or heightened anxiety. This item was also found to be the least meaningful indicator of the outburst factor. These issues align with a study by Remmert et al. (2022), which identified misspecifications in the original S-Five model relating to outburst. Remmert et al. (2022) proposed an alternative bifactor model that offers a clearer interpretation of the physical and verbal aspects of outbursts, which could potentially improve the item’s association with its intended *outburst* factor.

Finally, one item from the threat factor—'If I cannot get away from certain noises, I am afraid I might panic or feel like I will explode’—shared some conceptual similarity with the outburst dimension. This item was the only one from its original factor that emphasized a fear of explosion, which aligns with the element of the outburst factor about being afraid of doing something aggressive, while the remaining items in the threat factor focused on feelings of distress and helplessness when unable to avoid triggers.

These findings revealed the presence of three multidimensional items (out of 25 items) in the S-Five, which measured concepts from their intended theoretical dimensions while also showing some conceptual overlap with other aspects of misophonic experience. While these three items were found to be psychometrically sound for use in the S-Five, they could benefit from alterations to improve the stability of their respective dimensions.

### Cross-cultural comparisons of the S-Five factors

The highest average subscale score was for threat, which aligns with the findings from a study in the UK on individuals self-identifying with misophonia (Vitoratou et al., 2021b). Notably, when examining samples from the general population using the S-Five, other studies from the UK, Germany, China and Portugal report the highest average score on the externalizing factor (Remmert et al., 2022; Vitoratou et al., 2022; Hayes et al., 2024). When examining differences in the means on subscales between a misophonia sample and a general population sample, the average score is roughly 50% higher in a misophonia sample (compared to general population) for externalizing, but 3–4 times higher for the other four factors (Vitoratou et al., 2021b, 2023). While this group is heterogeneous in terms of diagnoses, with a majority experiencing misophonia, which could potentially influence the averages of the subscales, our results also show that externalizing did not correlate as strongly with the other four factors (0.26–0.38), when compared to intercorrelations between the other four (0.44–0.53). Externalizing also showed the lowest correlations with most other misophonia measures and with depression and anxiety in the present study and other studies (Vitoratou et al., 2021b; Remmert et al., 2022; Vitoratou et al., 2022, 2023; Hayes et al., 2024). While formal statistical comparisons were not used to examine these differences in this study, they contribute to the understanding of externalizing appraisals as relatively common responses to unpleasant sounds in the general population, and they may not be as useful as other factors in distinguishing misophonic responses from a general negative reaction to sounds. These outcomes also challenge the hypothesis (see for instance Norena, 2024) that misophonia is a result of negative attitudes towards certain behaviors or high moral standards, or a so-called ‘mental’ disorder, rooted in maladaptive beliefs. Nonetheless, such beliefs may probably intensify emotional reactions in misophonia. The issue of the impact of expectations and cultural norms warrants further exploration through cross-cultural studies, including qualitative investigations.

Average scores were lowest for the impact subscale, whereas in a UK misophonia sample, scores on the outburst subscale were lowest (Vitoratou et al., 2021b). Considering that the total S-Five score was higher in the current study (154 out of a possible 250) than the UK study (141 out of 250), it was surprising that the impact score was slightly lower for our Polish sample (21 out of 50) than in the UK sample (24 out of 50). The impact factor captures perceived current and future limitations caused by their reactions to sounds (for example, not seeing people you would like to see, or limited job opportunities), while the outburst factor measures aggression and worry about having aggressive outbursts. There may be cultural differences influencing these results. Research has found that social problem solving is more effective in Polish than British cultures (Kwaśniewska et al., 2014) and it is possible that this could result in less need to avoid situations where trigger sounds might be encountered, with more confidence that the problem could be resolved. Additionally, since the Polish sample reported more use of verbal aggression and shouting than the British sample, this may reflect a more reactive coping style than avoidant, compared to the British sample who scored lower on outburst items but higher on impact, which includes not seeing people and not going places one would otherwise like to see and go.

While shouting and verbal aggression were higher in the current study than in a similar sample in the UK (Vitoratou et al., 2021b), physical aggression and violence were two of the least endorsed statements. Fear of having an aggressive or violent outburst was rated much higher than actual physical aggression or violence, which is consistent with studies conducted in the UK (Vitoratou et al., 2021b), Germany (Remmert et al., 2022), China (Vitoratou et al., 2022), the Netherlands (Jager et al., 2020), and previous studies in Poland (Siepsiak et al., 2020b). It is also worth noting that outbursts showed stronger correlations with internalizing appraisals and threat than with externalizing appraisals, and outbursts were associated with anger, panic and distress reaction counts. This suggests that aggression in misophonia may be reflective of difficulties in regulating emotions in the presence of sounds, and is not necessarily about blaming or punishing the person making the sound. This is supported by a finding that impulse control on the Difficulties in Emotion Regulation Scale (DERS) was positively associated with misophonic outbursts, but not with externalizing appraisals (Remmert et al., 2022). There are differences across cultures in the psychological construct of aggression (Severance et al., 2013), so it is also possible that these differences could be explained by other variables not measured in the present study. Future research would benefit from distinguishing fear of potential aggressive outburst from actual aggressive behaviours, and looking at this in relation to emotion regulation skills in the context of misophonia and more generally, while also considering cultural norms in terms of shouting and verbal aggression. While there is some aggression reported in the presence of sounds, it is important that misophonia not be characterised as a disorder of violence, considering low reports of violence across cultures.

### Cross-cultural comparisons on the nature and intensity of trigger reactions

Eating sounds were reported as eliciting the most intense reactions, and repetitive environmental sounds among the least intense. This is consistent with other European studies using the S-Five (Vitoratou et al., 2021b; Remmert et al., 2022), but not consistent with a study in a Chinese population (Vitoratou et al., 2022), where other types of triggers (for instance, baby crying) evoked the strongest emotions. This suggests that there may be some cultural differences in the intensity of reactions to different sounds, or in the reporting of reactions.

In terms of the nature of the reaction to potential trigger sounds, no feeling and irritation were the most frequently reported reactions. While irritation and disgust are frequently reported as common emotions in misophonia (Schröder et al., 2017; Rouw and Erfanian, 2018; Kılıç et al., 2021; Guetta et al., 2022), our study found that the frequency of irritation and disgust were not positively associated with any of the S-Five factors, and in fact there was a low negative correlation between disgust and the outburst factor. This does not necessarily mean that irritation and disgust are not part of the experience of misophonia, but that as misophonia severity increases, we did not see a related increase in the reporting of irritation or disgust as the *primary* emotional reaction.

Irritation and disgust reaction counts were also not positively associated with the MisoQuest nor the MQ, apart from the MQ symptom subscale, which asks respondents the extent to which they are bothered by sounds compared to other people. Incidentally, this subscale had a low to moderate correlation with the S-Five, whereas the MisoQuest and other MQ subscales had strong positive correlations, suggesting that this specific subscale may be measuring a slightly different concept from the other misophonia scales.

These findings were consistent with a German study (Remmert et al., 2022), where irritation was not related to any of the S-Five factors, and disgust was only weakly associated with internalizing and externalizing appraisals. In UK studies, irritation was negatively correlated with S-Five total in a misophonia sample (Vitoratou et al., 2021b) and disgust was not associated with the S-Five except for a weak positive correlation with the threat factor. In a UK general population study, on the other hand, the S-Five had weak positive correlations with irritation and moderate positive correlations with disgust. A Chinese study also had weak positive correlations between irritation reaction counts and the S-Five, and disgust reaction counts were weakly associated with the threat subscale, but no other subscales (Vitoratou et al., 2022). In the Portuguese study, disgust was positively associated with all S-Five factors, albeit weakly, while irritation was weakly correlated with all factors except externalizing (Hayes et al., 2024).

Anger reaction counts were positively associated with all S-Five factors, which is consistent with other studies (Vitoratou et al., 2021b; Remmert et al., 2022; Vitoratou et al., 2022, 2023; Hayes et al., 2024). Panic and distress counts were positively associated with all factors except externalizing in the current study, but there are differences across other studies, with only the threat factor consistently associated with panic reactions, and only the threat and impact factors consistently related to distress reactions (Vitoratou et al., 2021b, 2022, 2023; Remmert et al., 2022; Hayes et al., 2024). Anger, panic and distress reaction counts have also been consistently related to total scores on MisoQuest (Remmert et al., 2022), the MQ (Vitoratou et al., 2021b, 2022, 2023; Hayes et al., 2024), and AMISO-S (Vitoratou et al., 2021b, 2022, 2023), with the exception of a Portuguese study where the A-MISO-S was associated with anger and panic, but not distress (Hayes et al., 2024).

Taken together, these results indicate that there is likely cross-cultural consistency in terms of the experience of anger, panic and distress reactions increasing in relation to overall misophonia severity. However, there is inconsistency across samples in terms of how those emotion reactions relate to specific aspects of misophonia, and several inconsistencies across samples in relation to the reactions of disgust and irritation. Anger, in particular, appears to be a core part of the misophonic experience across cultures.

These mixed findings across different studies may reflect some cultural differences in the experience (or reporting) of emotional reactions across cultures. There may be further differences in the relationship between emotion reaction counts and misophonia symptoms depending on the clinical status of the sample, which could be further tested with moderation analyses. Another possible explanation for differences in reactions to varying sounds across different studies could be related to non-cultural differences in the nature of the samples. Siepsiak et al. (2022) found that the prevalence of comorbid psychiatric disorders was significantly higher in the group of participants triggered by human oral sounds, than in those triggered by other, repetitive sounds. When comparing the present Polish sample with the Chinese sample (Vitoratou et al., 2022), there was a higher rate of reported co-occurrence of mental health conditions in the Polish sample (for example, 22% of the present sample reported depression, compared to 5% reporting depression in the Chinese sample). Future research would benefit from analyzing cross-cultural data together and controlling for co-occurring conditions.

Another interesting result, in line with a previous study in a German population (Remmert et al., 2022), is the low frequency of reporting physiological response to triggers, and no significant relationship between self-reported physiological reactions and misophonia symptoms. Considering that other studies have shown physiological reactions to sounds (Edelstein et al., 2013; Kumar et al., 2017; Siepsiak et al., 2023b), we propose that our findings do not imply an *absence* of physiological response. Participants were asked to provide one main response to each trigger, and while they may also experience physiological sensations, they may not consider this to be the primary response, or could see these sensations as part of their emotional response (for instance, attributing a racing heart to anger or panic). Additionally, many of the items in the S-Five contain an emotional element, and so for individuals who primarily recognize their physiological reactions rather than emotional (for example, those with alexithymia, who have difficulty recognizing and labelling emotions), they may not necessarily identify with the emotion-based wording of the S-Five items, which could contribute to the lack of association between the main S-Five and physiological reaction counts. More research into this would be helpful, given that recognition and management of psychophysiological reactions may be an important part of psychotherapeutic interventions (Lehrer, 2018). Future research would benefit from allowing multiple emotional and physiological response selections, using objective physiological measures, and incorporating a measure of alexithymia.

### Relationship between misophonia and anxiety sensitivity

While misophonia severity had a moderate association with current anxiety symptoms, it was only weakly associated with anxiety sensitivity. Internalizing appraisals demonstrated the highest correlation with anxiety sensitivity consistent with findings from German and UK samples (Remmert et al., 2022; Wang et al., 2022). In Portuguese study (Hayes et al., 2024), misophonia also exhibited a moderate association with anxiety symptoms but weak with anxiety sensitivity, with the highest correlation observed with threat.

Previous studies have found that anxiety sensitivity may explain some relationships between misophonia and other symptoms and emotion processes. For example, anxiety sensitivity mediated the relationship between core disgust and both misophonic distress and aggressive reactions (Barahmand et al., 2023). Anxiety sensitivity has also been found to moderate the relationship between misophonia and aggression (Schadegg et al., 2021), which is interesting considering our finding that only the cognitive subscale of anxiety sensitivity was associated with outbursts, and it was not associated with anger reaction counts. However, it should be noted that neither of these previous studies included current symptoms of anxiety in their models, which is also associated with higher anxiety sensitivity (Wheaton et al., 2012). In a previous study from our research group, anxiety sensitivity was not included in a final predictor model of misophonic outbursts, because exploratory models found that it was not a significant predictor of outbursts after accounting for anxiety symptoms (Wang et al., 2022). Therefore, future studies examining the role of anxiety sensitivity should also include a measure of current anxiety symptoms to better understand these relationships.

## Limitations

An important limitation of the study was the inability to test the five-factor structure identified through EFA using confirmatory factor analysis due to insufficient sample size. Our sample consisted primarily of individuals from online misophonia support groups, but it also included individuals who did not self-identify as having the condition. This enabled us to achieve a heterogenous sample for factor analysis, following recommendations by Gaskin et al. (2017). However, it means we could not establish scale norms for general or clinical populations. To ensure comprehensive validation, future studies should be done in both clinical and non-clinical samples, and within clinical samples, comparisons should be made between those with and without co-occurring conditions.

Additionally, diagnostic interviews for misophonia should be used in future studies to establish external validity and subclinical and clinical cut-off scores. Another limitation is that the current study did not test for discriminative validity with respect to other disorders of sound intolerance, namely tinnitus or hyperacusis, or neurodevelopmental conditions such as autism, attention deficit hyperactivity disorder (ADHD), traits of which are associated with sensory over-responsivity (Robertson and Simmons, 2013; Panagiotidi et al., 2018). Further work is needed to better understand the distinct features of misophonia as compared to conditions related to sound sensitivity and responsivity and disorders with similar emotional and behavioural processes. This could be done by using clinical comparison groups or by controlling for traits and symptoms that are potentially shared across disorders. Another significant limitation was recruiting participants from social media, which may have attracted non-representative misophonia sufferers with a particular psychological profile. This study represents the first psychometric validation in this population, and further validation studies using different and more controlled samples will be necessary.

Finally, the study was limited by its use of self-report of official diagnoses, including the information about the presence of co-occurring conditions. Future studies would benefit from using structured clinical interviews to examine the relationship between misophonia and other disorders more thoroughly in this cultural group.

## Conclusion

This study provided preliminary validation for the Polish version of the S-Five questionnaire, indicating the applicability and robustness of its five-factor structure in measuring misophonia symptoms within the Polish-speaking population. The tool can be utilized in clinical practice for diagnostic purposes and evaluating psychotherapeutic interventions, as well as in research, making it the first published tool in Polish to comprehensively assess misophonia severity with respect to its five components.

The study offers insights into misophonia’s nature, trigger sounds, emotional responses, and the association with depression, anxiety, and anxiety sensitivity, enhancing our understanding of the similarities and differences of this condition across varying cultures. Specifically, it highlights the significant role of anger, distress, and panic, while indicating the mixed role of irritation and disgust in misophonia across cultures. The findings emphasize that mouth sounds are more characteristic triggers of misophonia compared to other repetitive sounds, but that there are some cultural differences in the nature and intensity of reactions to various trigger sounds. These findings have important implications for further research and consideration of cultural differences in both research and the clinical management of misophonia.

## Data availability statement

The raw data supporting the conclusions of this article will be made available by the authors, without undue reservation.

## Ethics statement

The studies involving humans were approved by the King's College London Psychiatry, Nursing and Midwifery Research Ethics Subcommittee (REC Numer referencyjny: MOD-21/22-11826). The studies were conducted in accordance with the local legislation and institutional requirements. The participants provided their written informed consent to participate in this study.

## Author contributions

NU-M: Conceptualization, Formal analysis, Investigation, Methodology, Project administration, Validation, Writing – original draft, Writing – review & editing. MS: Funding acquisition, Investigation, Supervision, Writing – original draft, Writing – review & editing. JZ: Data curation, Project administration, Writing – original draft, Writing – review & editing. WD: Supervision, Writing – original draft, Writing – review & editing. JG: Supervision, Writing – original draft, Writing – review & editing. SV: Conceptualization, Investigation, Methodology, Software, Supervision, Writing – original draft, Writing – review & editing.
