# Supplementary material for: Beyond sound irritation: cross-cultural evidence on the robustness of the five aspects of misophonic experience measured by the S-Five in a Polish sample
Source: Front Psychol. 2024 Jun 19;15:1372870. doi: 10.3389/fpsyg.2024.1372870 (PMC11221307; doi:10.3389/fpsyg.2024.1372870)
Supplement: Supplementary file 1 [file Table_1.DOCX]

Supplementary Material 1

| **Table S1**. The S-Five in English and Polish. | |
| --- | --- |
| Please read each statement carefully and base your answer on how true they feel to you based on your current thoughts, experiences, and reactions: 0-not at all true to 10-completely true | Proszę uważnie przeczytać każdą pozycję i oprzeć swoją odpowiedź na tym, w jakim stopniu się z nimi utożsamiasz na podstawie aktualnych myśli, doświadczeń i reakcji: 0-zupełnie nieprawdziwe, do 10-całkowicie prawdziwe. |
|  |  |
| **Externalising** | **Eksternalizacja** |
| People should not make certain sounds, even if they do not know about others' sensitivities | Ludzie nie powinni wydawać niektórych dźwięków, nawet jeśli nie zdają sobie sprawy z nadwrażliwości innych osób na te dźwięki. |
| I get angry at other people because of how disrespectful they are with the noises they make | Złoszczę się na innych ludzi ze względu na ich brak szacunku wobec innych, gdy wydają pewne dźwięki. |
| People should do everything they can to avoid making noises that might bother others | Ludzie powinni robić wszystko, co w ich mocy, aby unikać wydawania dźwięków, które mogłyby przeszkadzać innym. |
| I react strongly to certain sounds because I cannot stand how selfish, thoughtless, or bad-mannered people can be | Reaguję silnie na niektóre dźwięki, ponieważ nie mogę znieść tego, jak samolubni, bezmyślni lub źle wychowani mogą być ludzie. |
| Certain sounds are just bad manners, and it is not strange to feel intense anger about that | Wydawanie niektórych odgłosów to po prostu złe maniery i to, że kogoś bardzo złości takie zachowanie, nie jest dziwne. |
| **Internalizing** | **Internalizacja** |
| The way I react to certain sounds makes me wonder whether deep inside I am just a bad person | Sposób w jaki reaguję na pewne dźwięki sprawia, że zastanawiam się, czy w głębi duszy jestem złą osobą. |
| The way I react to certain noises makes me feel like I must be an unlikable person deep down | Sposób w jaki reaguję na niektóre dźwięki sprawia, że w głębi duszy czuję, że ciężko mnie lubić. |
| I respect myself less because of my responses to certain sounds | Czuję do siebie mniej szacunku przez to, jak reaguję na pewne odgłosy. |
| I feel like I must be a very angry person inside because of the way I react to certain sounds | Czuję, że w głębi duszy muszę odczuwać dużą złość, skoro tak reaguję na niektóre dźwięki. |
| I dislike myself in the moments of my reactions to sounds | Nie lubię siebie w sytuacjach, gdy reaguję negatywnie na niektóre dźwięki. |
| **Impact** | **Wpływ na życie** |
| My job opportunities are limited because of my reaction to certain noises | Moje możliwości zatrudnienia są ograniczone ze względu na moje reakcje na niektóre dźwięki. |
| I do not meet friends as often as I would like to because of the noises they make | Czuję, że nie widuję przyjaciół tak często jak bym chciał/chciała z powodu dźwięków, jakie wydają. |
| There are places I would like to go but do not, because I am too worried about how the noises will impact me | Są miejsca do których chciałbym/abym pójść, ale tego nie robię, bo za bardzo martwię się tym, jak wpłyną na mnie niektóre dźwięki. |
| I can see future where I cannot do everyday things because of my reactions to noises | Patrząc w przyszłość, potrafię wyobrazić sobie sytuacje, gdy nie jestem w stanie wykonywać zwykłych codziennych czynności z powodu moich reakcji na dźwięki. |
| The way I feel/react to certain sounds will eventually isolate me and prevent me from doing everyday things | Sposób w jaki reaguję na niektóre dźwięki ostatecznie mnie odizoluje i uniemożliwi mi wykonywanie codziennych czynności. |
| **Outburst** | **Wybuchy emocji** |
| I can get so angry at certain noises that I get physically aggressive towards people to make them stop | Pewne dźwięki mogą mnie tak zezłościć, że staję się fizycznie agresywny/a w stosunku do ludzi, żeby tylko przestali je wydawać. |
| Sometimes I get so distressed by noises that I use violence to try and make it stop | Czasami niektóre dźwięki są źródłem takiej udręki, że używam przemocy, żeby spróbować je przerwać. |
| Some sounds are so unbearable that I will shout at people to make them stop | Niektóre dźwięki są tak nie do zniesienia, że zdarza mi się krzyczeć na ludzi, żeby przestali je wydawać. |
| If people make certain sounds that I cannot bear, I become verbally aggressive | Jeśli ludzie wydają pewne odgłosy, których nie mogę znieść, staję się słownie agresywny/a. |
| I am afraid I will do something aggressive or violent because I cannot stand the noise someone is making | Obawiam się, że mogę zacząć zachowywać się agresywnie i gwałtownie, gdy nie mogę znieść niektórych dźwięków, które ktoś wydaje. |
| **Threat** | **Zagrożenie** |
| I feel trapped if I cannot get away from certain noises | Czuję się osaczony/a, jeśli nie mogę uciec od niektórych dźwięków. |
| I feel anxious if I cannot avoid listening to certain sounds | Czuję duży niepokój, jeśli nie mogę uniknąć słuchania niektórych odgłosów. |
| If I cannot get away from certain noises, I am afraid I might panic or feel like I will explode | Jeśli nie mogę uciec od niektórych dźwięków, obawiam się, że wpadnę w panikę lub wybuchnę. |
| If I cannot avoid certain sounds, I feel helpless | Jeśli nie mogę uniknąć niektórych dźwięków, odczuwam bezradność. |
| I can experience distress as the result of some noises | Niektóre dźwięki stanowią dla mnie duży dyskomfort/są dla mnie udręką. |

| **Table S2.** The S-Five-T in English and Polish. | |
| --- | --- |
| Thinking about the past few weeks, what is the main feeling this sound* has caused you? *no feeling, irritation, distress, disgust, anger, panic, other feeling: negative, other feeling: positive, other: physiological reaction* | W ostatnich tygodniach, jakie uczucia najczęściej wywoływał w Tobie ten dźwięk*? żadne, irytacja, rozpacz, obrzydzenie, złość, panika, inne uczucie: negatywne, inne uczucie: pozytywne, inne: reakcję fizjologiczną |
| Thinking about the past few weeks, please rate the intensity of your reaction to this sound* when made by another person or object *(from 0: doesn't bother me at all to 10: unbearable/causes suffering)* | Oceń intensywność swojej reakcji na ten dźwięk* wydawany przez inną osobę lub obiekt, biorąc pod uwagę ostatnie kilka tygodni (*od 0: w ogóle mi nie przeszkadza do 10: nie do zniesienia/powoduje cierpienie*) |
| *List of triggers currently included in the S-Five-T: Normal eating sounds, Certain letter sounds, Mushy foods being eaten, Sound of clipping nails, Swallowing, Keyboard tapping, Lip smacking, Normal breathing, Repetitive engine noises, Loud/unusual breathing, Mobile phone sounds, Repetitive coughing, Humming noise, Repetitive sniffing, Snoring, Certain accents, Whistling sound, Sound of tapping, Rustling, Chewing gum, Footsteps, Hiccups, Slurping, Cutlery noises, Sneezing, Certain words, Kissing, Joint cracking, Muffled sounds, Throat clearing, Baby crying, Repetitive barking, Loud chewing, Clock ticking, Crunching eating sounds, Teeth sucking, Yawning. | *Lista stresorów/wyzwalaczy (ang. trigger) obecnie zawartych w S-Five-T: Normalne odgłosy jedzenia, Brzmienie niektórych głosek, Jedzenie papkowatych potraw, Dźwięk obcinania paznokci, Połykanie/przełykanie, Stukanie w klawiaturę, Mlaskanie, Normalne oddychanie, Odgłosy pracy silnika, Głośne/nietypowe oddychanie, Dźwięki telefonu komórkowego, Powtarzający się kaszel, Buczenie, Powtarzające się/ciągłe pociąganie nosem, Chrapanie, Niektóre rodzaje akcentu/intonacji mowy, Gwizdanie, Stukanie, Szeleszczący plastik lub papier, Głośne żucie gumy, Odgłosy kroków, Czkawka, Siorbanie, Odgłosy sztućców, Kichanie, Brzmienie niektórych słów, Odgłos pocałunków, Strzelanie stawów, Przytłumione dźwięki, Odchrząkiwanie, Płacz dziecka, Powtarzające się/ciągłe szczekanie, Głośne żucie, Tykanie zegara, Jedzenie chrupiących pokarmów, Zasysanie powietrza przez zęby/Syczenie, Ziewanie. |

| **Table S3.** Item stability values for the S-Five items across 1000 bootstrapped samples. | | | | | | | | |
| --- | --- | --- | --- | --- | --- | --- | --- | --- |
| **Item** | **Label** | **1** | **2** | **3** | **4** | **5** | **6** | **7** |
| int_S505 | Respect myself less | 0.999 |  |  | 0.001 |  |  |  |
| int_S518 | Bad person inside | 0.999 |  |  | 0.001 |  |  |  |
| int_S519 | Dislike self | 0.999 |  |  | 0.001 |  |  |  |
| int_S508 | Unlikeable person | 0.797 | 0.118 | 0.043 | 0.008 |  | 0.033 | 0.001 |
| int_S512 | Angry person inside | 0.614 | 0.213 | 0.122 | 0.006 | 0.001 | 0.044 |  |
| out_S517 | Physically aggressive | 0.016 | 0.981 |  | 0.003 |  |  |  |
| out_S522 | Violence | 0.016 | 0.981 |  | 0.003 |  |  |  |
| out_S523 | Shout at people | 0.017 | 0.978 | 0.002 | 0.002 |  | 0.001 |  |
| out_S504 | Verbally aggressive | 0.049 | 0.875 | 0.052 | 0.003 |  | 0.021 |  |
| out_S524 | Afraid of outburst | 0.198 | 0.621 | 0.128 | 0.001 | 0.001 | 0.051 |  |
| thr_S507 | Feel anxious | 0.003 | 0.001 | 0.996 |  |  |  |  |
| thr_S511 | Feel trapped | 0.003 | 0.001 | 0.996 |  |  |  |  |
| thr_S510 | Experience distress | 0.003 | 0.002 | 0.995 |  |  |  |  |
| thr_S503 | Feel helpless | 0.004 | 0.002 | 0.994 |  |  |  |  |
| thr_S502 | Panic or explode | 0.091 | 0.219 | 0.662 | 0.004 |  | 0.024 |  |
| imp_S501 | Do not meet friends |  |  |  | 1.000 |  |  |  |
| imp_S514 | Avoid places |  |  |  | 1.000 |  |  |  |
| imp_S520 | Limited job opportunities |  |  |  | 1.000 |  |  |  |
| imp_S515 | Cannot do everyday things |  |  |  | 0.999 |  | 0.001 |  |
| imp_S509 | Eventually isolated | 0.028 | 0.012 | 0.007 | 0.946 |  | 0.006 | 0.001 |
| ext_S506 | Others avoid making noises |  |  |  |  | 1.000 |  |  |
| ext_S513 | Others should not make sounds |  |  |  |  | 1.000 |  |  |
| ext_S516 | Others selfish |  |  |  |  | 1.000 |  |  |
| ext_S521 | Others bad manners |  |  |  |  | 1.000 |  |  |
| ext_S525 | Others disrespectful |  |  |  |  | 1.000 |  |  |
| *Note.* 1: Internalising factor; 2: Outburst factor; 3: Threat factor; 4: Impact factor; 5: Externalising factor; 6-7: factors identified in bootstrap EGA that had a low proportion of replications and were not assigned a name.  Blank cells correspond to 0. | | | | | | | | |
