# Supplementary material for: Beyond sound irritation: cross-cultural evidence on the robustness of the five aspects of misophonic experience measured by the S-Five in a Polish sample
Source: Front Psychol. 2024 Jun 19;15:1372870. doi: 10.3389/fpsyg.2024.1372870 (PMC11221307; doi:10.3389/fpsyg.2024.1372870)
Supplement: Supplementary file 2 [file Data_Sheet_1.pdf]

S-Five

Imię/Identyfikator:

Data:

Proszę uważnie przeczytać każdą pozycję i oprzeć swoją odpowiedź na tym, w jakim stopniu się z nimi utożsamiasz na podstawie aktualnych <u>myśli, doświadczeń i reakcji</u>: 0-zupełnie nieprawdziwe, do 10-całkowicie prawdziwe.

Zupełnie nieprawdziwe — **Całkowicie prawdziwe**

	0	1	2	3	4	5	6	7	8	9	10
**1.**	**Ludzie nie powinni wydawać niektórych dźwięków, nawet jeśli nie zdają sobie sprawy z nadwrażliwości innych osób na te dźwięki.**										
	○	○	○	○	○	○	○	○	○	○	○
**2.**	**Sposób w jaki reaguję na niektóre dźwięki sprawia, że w głębi duszy czuję, że ciężko mnie lubić.**										
	○	○	○	○	○	○	○	○	○	○	○
**3.**	**Są miejsca do których chciałbym/abym pójść, ale tego nie robię, bo za bardzo martwię się tym, jak wpłyną na mnie niektóre dźwięki.**										
	○	○	○	○	○	○	○	○	○	○	○
**4.**	**Czuję do siebie mniej szacunku przez to, jak reaguję na pewne odgłosy.**										
	○	○	○	○	○	○	○	○	○	○	○
**5.**	**Czuję się osaczony/a, jeśli nie mogę uciec od niektórych dźwięków.**										
	○	○	○	○	○	○	○	○	○	○	○
**6.**	**Jeśli nie mogę uniknąć niektórych dźwięków, odczuwam bezradność.**										
	○	○	○	○	○	○	○	○	○	○	○
**7.**	**Sposób w jaki reaguję na pewne dźwięki sprawia, że zastanawiam się, czy w głębi duszy jestem złą osobą.**										
	○	○	○	○	○	○	○	○	○	○	○
**8.**	**Niektóre dźwięki są tak nie do zniesienia, że zdarza mi się krzyczeć na ludzi, żeby przestali je wydawać.**										
	○	○	○	○	○	○	○	○	○	○	○
	0	1	2	3	4	5	6	7	8	9	10

Zupełnie nieprawdziwe — **Całkowicie prawdziwe**

Zupełnie nieprawdziwe **Całkowicie prawdziwe**

	0	1	2	3	4	5	6	7	8	9	10
9.	Niektóre dźwięki stanowią dla mnie duży dyskomfort/są dla mnie udręką.										
	○	○	○	○	○	○	○	○	○	○	○
10.	Moje możliwości zatrudnienia są ograniczone ze względu na moje reakcje na niektóre dźwięki.										
	○	○	○	○	○	○	○	○	○	○	○
11.	Wydawanie niektórych odgłosów to po prostu złe maniery i to, że kogoś bardzo złości takie zachowanie, nie jest dziwne.										
	○	○	○	○	○	○	○	○	○	○	○
12.	Jeśli ludzie wydają pewne odgłosy, których nie mogę znieść, staję się słownie agresywny/a.										
	○	○	○	○	○	○	○	○	○	○	○
13.	Złoszczę się na innych ludzi ze względu na ich brak szacunku wobec innych, gdy wydają pewne dźwięki.										
	○	○	○	○	○	○	○	○	○	○	○
14.	Sposób w jaki reaguję na niektóre dźwięki ostatecznie mnie odizoluje i uniemożliwi mi wykonywanie codziennych czynności.										
	○	○	○	○	○	○	○	○	○	○	○
15.	Pewne dźwięki mogą mnie tak zezłościć, że staję się fizycznie agresywny/a w stosunku do ludzi, żeby tylko przestali je wydawać.										
	○	○	○	○	○	○	○	○	○	○	○
16.	Ludzie powinni robić wszystko, co w ich mocy, aby unikać wydawania dźwięków, które mogłyby przeszkadzać innym.										
	○	○	○	○	○	○	○	○	○	○	○
17.	Jeśli nie mogę uciec od niektórych dźwięków, obawiam się, że wpadnę w panikę lub wybuchnę.										
	○	○	○	○	○	○	○	○	○	○	○
	0	1	2	3	4	5	6	7	8	9	10

Zupełnie nieprawdziwe **Całkowicie prawdziwe**

Zupełnie nieprawdziwe **Całkowicie prawdziwe**

	0	1	2	3	4	5	6	7	8	9	10
18.	**Czuję duży niepokój, jeśli nie mogę uniknąć słuchania niektórych odgłosów.**										
	○	○	○	○	○	○	○	○	○	○	○
19.	**Czasami niektóre dźwięki są źródłem takiej udręki, że używam przemocy, żeby spróbować je przerwać.**										
	○	○	○	○	○	○	○	○	○	○	○
20.	**Czuję, że nie widuję przyjaciół tak często jak bym chciał/a z powodu dźwięków, jakie wydają.**										
	○	○	○	○	○	○	○	○	○	○	○
21.	**Czuję, że w głębi duszy muszę odczuwać dużą złość, skoro tak reaguję na niektóre dźwięki.**										
	○	○	○	○	○	○	○	○	○	○	○
22.	**Reaguję silnie na niektóre dźwięki, ponieważ nie mogę znieść tego, jak samolubni, bezmyślni lub źle wychowani mogą być ludzie.**										
	○	○	○	○	○	○	○	○	○	○	○
23.	**Nie lubię siebie w sytuacjach, gdy reaguję negatywnie na niektóre dźwięki.**										
	○	○	○	○	○	○	○	○	○	○	○
24.	**Patrząc w przyszłość, potrafię wyobrazić sobie sytuacje, gdy nie jestem w stanie wykonywać zwykłych codziennych czynności z powodu moich reakcji na dźwięki.**										
	○	○	○	○	○	○	○	○	○	○	○
25.	**Obawiam się, że mogę zacząć zachowywać się agresywnie i gwałtownie, gdy nie mogę znieść niektórych dźwięków, które ktoś wydaje.**										
	○	○	○	○	○	○	○	○	○	○	○
	0	1	2	3	4	5	6	7	8	9	10

Zupełnie nieprawdziwe **Całkowicie prawdziwe**

Wynik ogólny	Eksternalizacja	Internalizacja	Wpływ na życie	Wybuchy emocji	Zagrożenie
0	0	0	0	0	0
0-250	0-50	0-50	0-50	0-50	0-50

Jeśli nie widzisz automatycznie obliczonych sum, upewnij się, że nie pominąłeś żadnego z pytań. W przeciwnym przypadku możliwe jest, że używasz niekompatybilnej przeglądarki PDF lub edytowanej wersji kwestionariusza, w której usunięto kod JavaScript odpowiedzialny za obliczenia.

Usuń wszystkie dane z formularza

Uglik-Marucha N, Siepsiak M, Zielińska J, Dragan WŁ, Gregory J and Vitoratou S. (2024). Beyond sound irritation: cross-cultural evidence on the robustness of the five aspects of misophonic experience measured by the S-Five in a Polish sample. Front. Psychol. 15:1372870. doi: 10.3389/fpsyg.2024.1372870

Kontakt:
Nora Uglik-Marucha
eleonora.uglik-marucha@kcl.ac.uk
